# Engineered *Lactobacillus paracasei* 3×pBD1/*ΔHLJ-27* as a novel antibiotic alternative for livestock: Dual efficacy in growth promotion and protection against *Salmonella* infection via integrated host-microbiota-immune regulation

**DOI:** 10.1371/journal.ppat.1014367

**Published:** 2026-08-14

**Authors:** Yijie Yang, Ying Chen, Haiyuan Zhao, Zhaojun Wang, Meijun Yu, Xijiao Shao, Yin Zhu, Ping Han, Tianyu Han, Wen Cui, Yanping Jiang, Lijie Tang, Yijing Li, Jiaxuan Li, Xiaona Wang

**Affiliations:** 1 College of Veterinary Medicine, Northeast Agricultural University, Harbin, China; 2 Heilongjiang Key Laboratory for Animal Disease Control and Pharmaceutical Development, Harbin, China; University of the Chinese Academy of Sciences, CHINA

## Abstract

To address the antimicrobial resistance (AMR) crisis driven by livestock agriculture, effective antibiotic alternatives are urgently needed. However, current probiotic and antimicrobial peptide strategies are critically hindered by limited efficacy, poor stability, and a lack of systemic protection. This study employed recombinant *Lactobacillus paracasei* 3 × pBD1/*ΔHLJ-27*, which can secrete and express multicopy porcine β-defensin-1 (pBD1), a cationic antimicrobial peptide with broad-spectrum activity. The study demonstrate that this engineered strain confers systemic protection against *Salmonella* Typhimurium (*S*. Typhimurium) infection by coordinately enhancing intestinal barrier integrity and modulating host immune responses. Specifically, 3 × pBD1/*ΔHLJ-27* improved intestinal morphology and upregulated tight junction proteins, while promoting balanced immune activation characterized by increased CD4 ⁺ T cells and elevated SIgA and IgG levels. In infection models, the strain significantly reduced mortality, limited bacterial dissemination, and alleviated intestinal pathology. Mechanistically, these protective effects are associated with suppression of excessive inflammatory responses and reprogramming of host-microbiota interactions, as evidenced by reduced pro-inflammatory signaling and enrichment of beneficial microbial taxa. Collectively, these findings identify 3 × pBD1/*ΔHLJ-27* as a multifunctional engineered probiotic that integrates antimicrobial activity with immune regulation, offering a promising and scalable alternative to antibiotics in animal production.

## Author summary

Antimicrobial resistance (AMR) is a global public health challenge within the One Health framework. Current alternative strategies in animal agriculture, including probiotics and antimicrobial peptides, are limited by suboptimal efficacy, instability, and poor systemic distribution. Here, we engineered a recombinant *Lactobacillus paracasei* 3 × pBD1/*ΔHLJ-27* that constitutively expresses multicopy porcine β-defensin-1 (pBD1), a cationic antimicrobial peptide with broad-spectrum activity, and systematically evaluated its potential as a multifunctional antibiotic alternative in livestock settings. Our research demonstrates that this strain not only promotes animal growth by enhancing body weight and stimulating the development of intestinal villi, but also improves intestinal barrier function by increasing the expression of tight junction proteins. Additionally, it boosts both systemic and local immune responses by increasing immune organ size, raising CD4^+^ T cell populations, and enhancing immunoglobulin levels. In *S.* Typhimurium*-*infection models, the 3 × pBD1/*ΔHLJ-27* drastically reduces mortality rates, curbs systemic bacterial dissemination, and attenuates intestinal pathological damage, while concurrently reshaping the gut microbiota toward a more anti-inflammatory and pathogen-resistant composition. Collectively, the 3 × pBD1/*ΔHLJ-27* as a first-in-class engineered probiotic that integrates antimicrobial activity, gut barrier protection, immune modulation, and microbiota regulation-addressing the longstanding limitations of single-function antibiotic alternatives.

## Introduction

*Salmonella,* a major food-borne zoonotic pathogen, infects a broad spectrum of animal hosts and humans, commonly inducing fever, vomiting and diarrhea, with severe infections potentially progressing to life-threatening systemic sepsis [1]. Non-typhoidal salmonellosis caused by predominant zoonotic serovars of *Salmonella* enterica, notably *Salmonella* Typhimurium, imposes a substantial and growing public-health burden in China [2]. Systematic reviews and meta-analyses have demonstrated that non-typhoidal salmonellosis caused by predominant zoonotic serovars of *Salmonella* enterica, notably *S.* Typhimurium, imposes a substantial and growing public-health burden across China under the One-Health framework [3]. Furthermore, nationwide epidemiological surveillance has revealed that the temporal dynamics of circulating resistant strains are increasingly driven by rising antimicrobial resistance in this region [4–6]. Recent Chinese genomic surveillance and retrospective epidemiological analyses further demonstrate that zoonotic *Salmonella* strains, particularly invasive *S.* Typhimurium ST313 lineages, readily acquire resistance in livestock reservoirs and transmit to humans via the food chain, severely undermining clinical treatment efficacy [7,8]. Against this backdrop, developing non-antibiotic alternatives such as probiotic-based interventions has become an urgent public-health priority.

Antimicrobial peptides (AMPs) play essential roles in host defense against microbial infection, growth-promoting effects, and immunomodulatory characteristics [9–11]. Defensins are a category of cationic AMPs distinguished by their small molecular size (2–5 kDa) and elevated cysteine content [12]. Porcine β-defensin 1 (pBD1), secreted by porcine epithelial cells, is a critical component of the host’s innate immune system [13]. It exerts broad-spectrum inhibitory effects against bacteria, fungi, parasites, and viruses, exhibits low propensity for inducing drug resistance, and confers a growth-promoting effect on the organism [13–15]. Recent reports have suggested that the defensins are vital for maintaining intestinal homeostasis and restoring the gut microbiota [16].

Defensins face significant obstacles in large-scale manufacture and clinical use due to their naturally modest expression levels, together with the technical intricacies and elevated costs linked to *in vitro* synthesis. Lactic acid bacteria (LAB), acknowledged as food-grade microorganisms [17], demonstrate many probiotic attributes such as the regulation of gut microbiota equilibrium, inhibition of pathogenic microbial colonization, and improvement of intestinal immune function. Notably, specific *Lactobacillus* consortia have been shown to possess remarkable efficacy in recovering microbiome diversity and restoring immune homeostasis, even under severe physiological stress such as chemotherapy-induced damage [18–21]. *Lactobacillus* display significant resilience to severe gastrointestinal conditions, such as gastric acidity, bile salts, and intestinal enzymes, while also exhibiting enhanced mucosal adhesion properties [22,23]. Furthermore, specific LAB formulations have demonstrated significant resilience to metabolic stressors, such as hyperlipidemia and cholesterolemia [24]. While broad-spectrum treatments traditionally rely on complex multi-strain probiotic “cocktails” [24], single engineered *Lactobacillus* systems provide a more streamlined and controllable strategy. The versatility of these engineered platforms extends far beyond enteric infections to advanced systemic treatments, including cancer therapy [25]. These integrated characteristics establish ideal conditions for effective gut colonization and sustained probiotic function. Thus, the technological advancement of *Lactobacillus*-based delivery systems has arisen as a strategic answer [26–28].

Consequently, LAB represent an ideal oral mucosal delivery vector. Utilizing them to express defensins not only circumvents the traditional bottlenecks of defensin production and clinical application, but also synergistically amplifies the inherent probiotic efficacy of the bacteria. Due to defensins being short peptide genes, a multi-copy strategy can enhance their expression, and numerous studies have effectively produced high-yield proteins using this approach [29]. Lee et al. [30] constructed a multicopy expression vector for the cationic antimicrobial peptide buforin II by fusing the target gene with an anionic peptide gene fragment and placing the fusion sequence under the control of the tac promoter. Using the complete operon containing the buforin II gene as a tandem unit, they assembled a multicopy expression vector, which was successfully expressed in *Escherichia coli* BL21. Pastoris.Mansu et al. [31] constructed a multicopy proinsulin expression cassette in Pichia pastoris using a compatible cohesive-end cloning strategy. The strain harboring 11 gene copies achieved a proinsulin expression level of 250 mg/L, which was significantly higher than the 19 mg/L obtained with the single-copy construct. The advancement of live vector vaccines capable of promoting exogenous defensin expression has emerged as a vital research focus.

Previous studies have confirmed that wild-type *Lactobacillus paracasei* (*L. paracasei*) exerts anti-*Salmonella* effects via regulating intestinal immunity, inhibiting pathogen colonization and virulence gene expression, and alleviating infection-induced inflammation [32,33]. In this study, we constructed the engineered strain capable of secreting multi-copy porcine pBD1, which integrates the inherent probiotic properties of lactic acid bacteria with the broad-spectrum antibacterial and immunomodulatory activities of pBD1. Its rational combination with wild-type *L. paracasei* achieves synergistic protective effects. Compared with traditional single-strain probiotic applications, our synergistic combination strategy is a key innovation of this research. This research offers essential scientific data for the development of a novel oral *Lactobacillus* formulation through assessment of both *in vitro* antibacterial activity and *in vivo* protective effects after oral administration. This formulation possesses considerable potential for practical applications in ef ficiently avoiding intestinal bacterial infections and augmenting host innate immune capabilities in livestock production.

## Results

### Effects of oral administration of recombinant strain on the growth performance of mice and piglets

**Fig 1:**
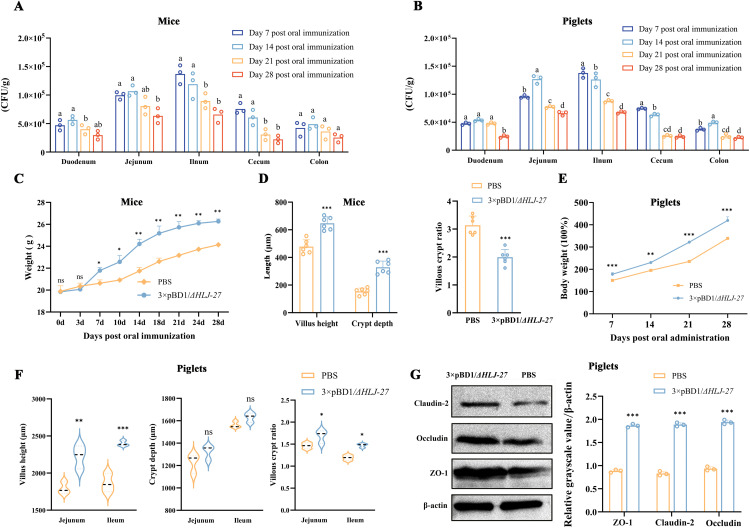
3 × pBD1/*ΔHLJ-27* colonizes the intestine to improve growth and barrier integrity in mice and piglets. **A**, **B**. Plate assay-based identification of 3 × pBD1/*ΔHLJ-27* in intestinal mucus of mice and piglets. **C**. The body weight of the mice with different treatments. **D**. Assessment of villus length, crypt depth, and the villus-crypt ratio in the small intestine of mice. **E**. The body weight of the piglets with different treatments. **F**. Assessment of villus length, crypt depth, and the villus-crypt ratio in the small intestine of piglets. **G**. Western blot analysis of tight junction protein expression in ileum epithelium of piglets. Note: Data are presented as mean ± SD; n = 6 per group in mice, n = 3 per group in piglets. Different letters (a vs. b, a vs. c, a vs. d, b vs. c, b vs. d, c vs. d) denote statistically significant differences (*P* < 0.05). ^*^P < 0.05, ^**^P < 0.01, ^***^P < 0.001 vs PBS.

The specific oral administration program for mice and piglets is shown in S1A and S1D Fig. The recombinant strain 3 × pBD1/*ΔHLJ-27* demonstrated robust intestinal colonization following oral administration in both mouse and neonatal piglet models. In mice, the strain colonized the entire intestinal tract from the duodenum to the colon, with the highest bacterial density observed in the jejunum and ileum (Figs 1A and S1B). Mice following oral administration showed significantly greater body weight than PBS-treated controls from day 7 onward, without mortality or signs of illness (Fig 1C). Histological examination of the ileum revealed intact epithelial structures in both groups, while the recombinant strain group exhibited increased villus height, crypt depth, and villus-to-crypt ratio, suggesting enhanced intestinal barrier integrity (Figs 1D and S1C). Similarly, stable colonization was observed in neonatal piglets, becoming evident by day 7, peaking at day 14, and persisting until day 28 with a slight decline after day 21 (Figs 1B and S1E). Piglets orally administered 3 × pBD1/*ΔHLJ-27* exhibited significantly greater weight gain compared with PBS controls. (Fig 1E). Histological analysis of the jejunum and ileum showed preserved intestinal morphology in both groups, whereas the recombinant strain group displayed increased villus height and villus-to-crypt ratio (Figs 1F and S1F). Consistently, Western blot analysis revealed elevated expression of tight junction proteins ZO-1, Occludin, and Claudin-2 in the recombinant strain group (Fig 1G), indicating improved intestinal barrier function.

**Fig 2:**
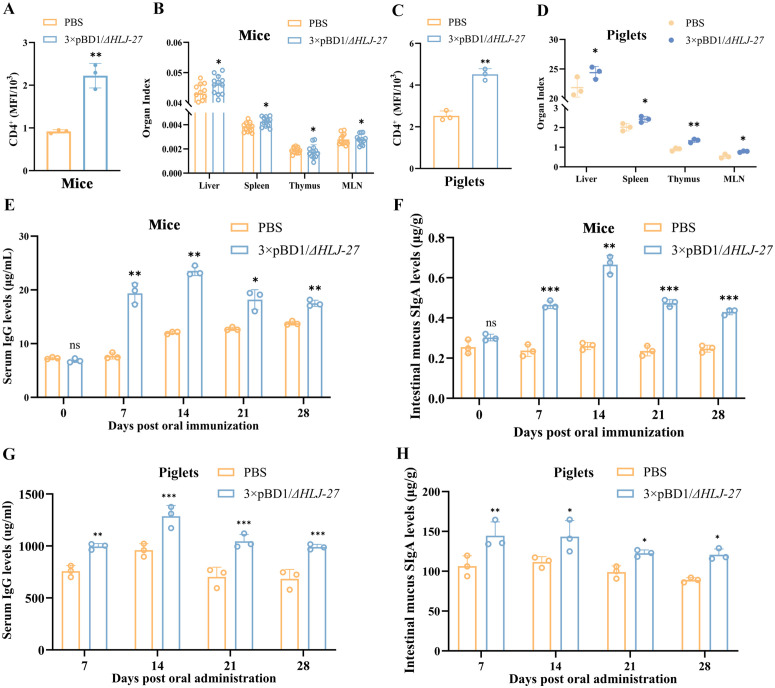
Systemic and mucosal immune enhancement by 3 × pBD1/*ΔHLJ-27.* Detection of CD4 ⁺ T cell content in mesenteric lymph nodes of mice. (**A**) and piglets **(C)**. Effects of the recombinant strain 3 × pBD1/*ΔHLJ-27* on organ index in mice (**B**) and piglets **(D)**. Effects of oral treatment of recombinant strain 3 × pBD1/*ΔHLJ-27* on blood IgG and intestinal mucosal SIgA levels in mice (**E, F**) and piglets **(G, H)**. Note: Data are presented as mean ± SD; n = 3 per group. ^*^P < 0.05, ^**^P < 0.01, ^***^P < 0.001 vs PBS.

Flow cytometry revealed a significantly higher proportion of CD4 ⁺ T cells in the mesenteric lymph nodes of both mice and piglets treated with 3 × pBD1/*ΔHLJ-27*, indicating activation of gut-associated immune responses (Fig 2A and 2C). Correspondingly, the recombinant strain significantly increased the immune organ indices of the liver, spleen, thymus, and mesenteric lymph nodes in both animal models compared with PBS controls (Fig 2B and 2D). Consistently, serum IgG and intestinal mucosal SIgA levels were significantly elevated following oral administration of 3 × pBD1/*ΔHLJ-27*, beginning at day 7, peaking at day 14, and remaining higher than those in the control group thereafter in both mice and piglets (Fig 2E-H). Together, these findings demonstrate that 3 × pBD1/*ΔHLJ-27* promotes immune organ development and enhances both systemic humoral and mucosal immune responses.

### Efficacy of oral recombinant strain against intestinal pathogenic bacteria infection in mice

**Fig 3:**
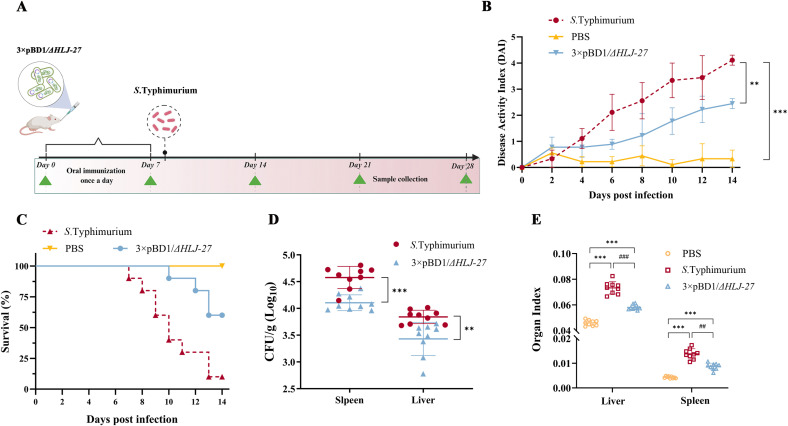
Efficacy of oral recombinant strain against intestinal *S.* Typhimurium infection in mice. **A**. Illustration of the immunoprotective protocol involving recombinant strain 3 × pBD1/*ΔHLJ-27* in mice subjects. **B**. The DAI score of mice. **C**. Survival curves of mice infected with ***S.*** Typhimurium*.* **D**. The number of ***S.*** Typhimurium in mice tissues. **E**. Determination of organ index in mice infected with ***S.*** Typhimurium. Fig 3A created in BioRender. Yang, **Y. Y.** (2026) https://BioRender.com/zknauu4. Note: Data are presented as mean ± SD; n = 9 per group in mice. ^*^P < 0.05, ^**^P < 0.01, ^***^P < 0.001 vs ***S.*** Typhimurium.

The specific oral administration schedule for the study examining the anti-infection effects of oral recombinant strain in mice is illustrated in Fig 3A. Mice infected with *S.* Typhimurium exhibited a continuous increase in DAI scores beginning on day 4 (Fig 3B), indicating a progressive deterioration of clinical symptoms. In contrast, mice pretreated with the recombinant strain 3 × pBD1/*ΔHLJ-27* demonstrated a significant reduction in illness severity compared to the infected controls. Importantly, this alleviation of macroscopic clinical symptoms directly translated into survival rate; while the survival rate of the infected group plummeted to 10%, the oral administration of 3 × pBD1/*ΔHLJ-27* robustly rescued the survival rate to 60% (Fig 3C). To elucidate the underlying basis of this protective effect, we quantified the systemic bacterial burden. Consistent with the improved clinical outcomes, mice treated with 3 × pBD1/*ΔHLJ-27* displayed a marked reduction in *S.* Typhimurium colonization within the liver and spleen (Fig 3D), demonstrating that the recombinant strain effectively restricts bacterial dissemination and bolsters host resistance. Mice infected with *S.* Typhimurium demonstrated increased liver and spleen indices. Oral administration of the recombinant strain 3 × pBD1/*ΔHLJ-27* resulted in a reduction of organ enlargement, suggesting a protective effect against *S.* Typhimurium-induced damage (Fig 3E).

**Fig 4:**
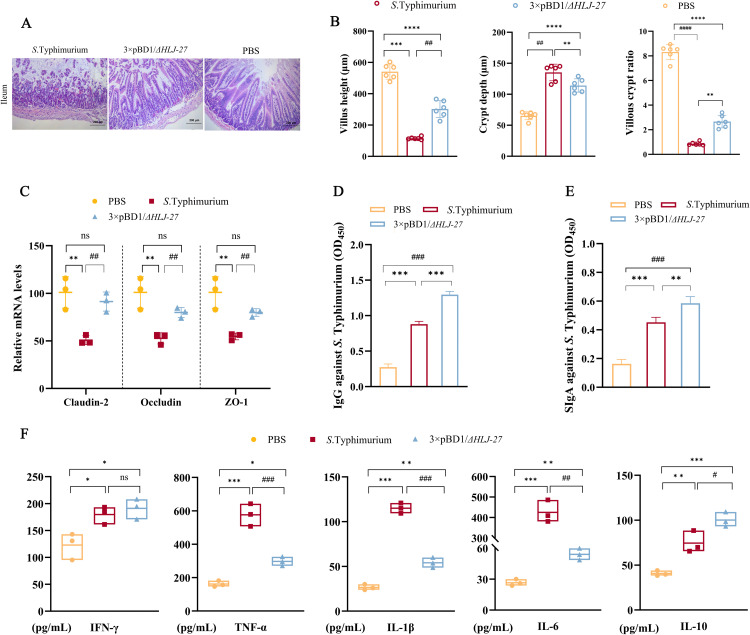
Recombinant strain improves intestinal barrier function and modulates immune responses during *S.* Typhimurium infection in mice. **A**. Effect of 3 × pBD1/*ΔHLJ-27* on ileum morphology. **B**. Assessment of the villi length, crypt depth and the villus-crypt ratio in the ileum intestine. **C**. Real-time PCR analysis of tight junction protein expression in ileum of mice. **D, E**. Serum-specific IgG and intestinal-specific SIgA levels against ***S.*** Typhimurium. **F**. Determination of inflammatory secretion level of mice. Note: Data are presented as mean ± SD; n = 3 per group. ^*^P < 0.05, ^**^P < 0.01, ^***^P < 0.001 vs ***S.*** Typhimurium; ^#^*P* < 0.05, ^##^*P* < 0.01, ^###^*P* < 0.001, 3 × pBD1/*ΔHLJ-27* vs pPG/*ΔHLJ-27*; ns, *P* > 0.05.

To evaluate the protective effect of 3 × pBD1/*ΔHLJ-27* on intestinal integrity, histopathological analysis was performed using H&E staining (Fig 4A). While the PBS control group displayed typical intestinal morphology with intact villous structures, mice infected with *S.* Typhimurium exhibited severe ileal mucosal damage, characterized by villous atrophy, significant submucosal edema, and substantial inflammatory infiltration. In contrast, oral administration of the recombinant strain 3 × pBD1/*ΔHLJ-27* markedly ameliorated these pathological changes, preserving relatively intact villous structures with minimal edema. Consistent with these observations, treatment with 3 × pBD1/*ΔHLJ-27* significantly restored villus height, crypt depth, and the villus-to-crypt ratio, with values approaching those of the PBS control group (Fig 4B). Furthermore, RT-qPCR analysis revealed that *S.* Typhimurium infection severely downregulated the mRNA expression of critical tight junction proteins (ZO-1, Claudin-2, and Occludin) in the ileum; however, this reduction was effectively reversed by 3 × pBD1/*ΔHLJ-27* pretreatment (Fig 4C). These results demonstrate that the recombinant strain effectively mitigates infection-induced intestinal injury and maintains barrier integrity.

Immunological analysis further confirmed the efficacy of 3 × pBD1/*ΔHLJ-27* in boosting host defense. Compared to the infected control group, mice treated with the recombinant strain displayed significantly elevated levels of *S.* Typhimurium-specific IgG and SIgA antibodies, indicating robust systemic and mucosal immune responses (Fig 4D and 4E). Cytokine profiling (Fig 4F) revealed a distinct immune-modulatory effect induced by 3 × pBD1/*ΔHLJ-27*. While *S.* Typhimurium infection triggered a severe inflammatory response, characterized by sharply elevated serum levels of pro-inflammatory cytokines (IFN-γ, IL-1β, IL-6, and TNF-α), treatment with the recombinant strain significantly attenuated the overproduction of these pro-inflammatory mediators. Concurrently, this reduction in inflammation was accompanied by a marked elevation in the anti-inflammatory cytokine IL-10. This distinct profile suggests that 3 × pBD1/*ΔHLJ-27* effectively shifts the immune response towards a balanced, anti-inflammatory state, mitigating hyper-inflammation while maintaining necessary host defenses.

### Mechanistic study of recombinant strain 3 × pBD1/*ΔHLJ-27* in modulating infectious progression and intestinal barrier repair in piglets

**Fig 5:**
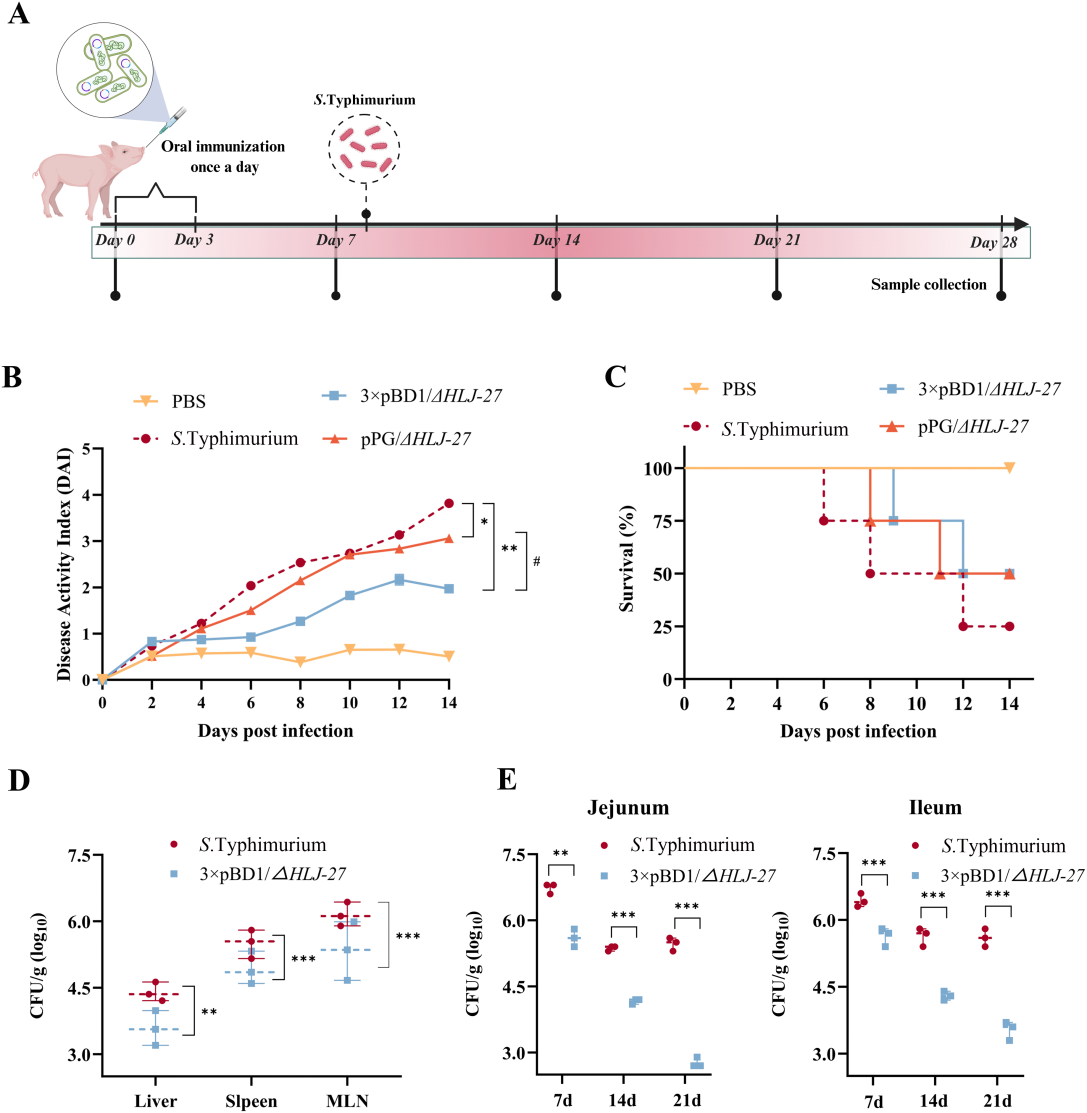
Protective efficacy and bacterial clearance in *S.* Typhimurium-infected piglets following administration with the recombinant strain 3 × pBD1/*ΔHLJ-27* in piglets. **A**. Illustration of the immunoprotective protocol involving recombinant strain 3 × pBD1/*ΔHLJ-27* in piglets subjects. **B**. The DAI of piglets during the oral administration cycle. **C**. Survival curves of piglets infected with ***S.*** Typhimurium*.* **D**. Quantification of ***S.*** Typhimurium burdens in the Liver, Spleen, and MLN of challenged piglets. **E**. ***S.*** Typhimurium loads in the jejunum and ileum mucosa at 7, 14, and 21 days post-infection. Fig 5A created in BioRender. Yang, **Y. Y.** (2026) https://BioRender.com/cx2hxnp. Note: Data are presented as mean ± SD; n = 3 per group. ^*^P < 0.05, ^**^P < 0.01, ^***^P < 0.001 vs ***S.*** Typhimurium, ns, *P* > 0.05. ^#^*P* < 0.05, 0.01 < ^##^*P* < 0.05, ^###^*P* < 0.01, 3 × pBD1/*ΔHLJ-27* vs pPG/*ΔHLJ-27*; ns, *P* > 0.05.

In the *S.* Typhimurium infection experiment, conducted following a 3-day oral administration protocol (Fig 5A), it was observed that both the recombinant strain 3 × pBD1/*ΔHLJ-27* and the pPG-PPT/*ΔHLJ-27* groups significantly decreased disease activity indices in comparison to the *S.* Typhimurium-infected group, which displayed severe clinical symptoms. Importantly, the 3 × pBD1/*ΔHLJ-27* strain exhibited superior protective efficacy (Fig 5B). The survival rates were markedly higher in the recombinant 3 × pBD1/*ΔHLJ-27* group, achieving 60%, as opposed to 20% in the *S.* Typhimurium-infected group and 40% in the empty vector control group (Fig 5C).

The mucosal immune response of newborn piglets to *S.* Typhimurium infection was enhanced by oral administration of recombinant strain 3 × pBD1/*ΔHLJ-27*. The recombinant strain 3 × pBD1/*ΔHLJ-27* significantly inhibited the spread and colonization of *S.* Typhimurium, as evidenced by significantly reduced bacterial loads in the liver, spleen, and mesenteric lymph nodes when compared to the infected group (Fig 5D). Compared with piglets infected with *S.* Typhimurium, pretreatment with the recombinant strain significantly reduced bacterial loads in the jejunal and ileal mucosa. In addition, the intestinal *S.* Typhimurium burden progressively decreased over time at 7, 14, and 21 days post-infection (Fig 5E).

**Fig 6:**
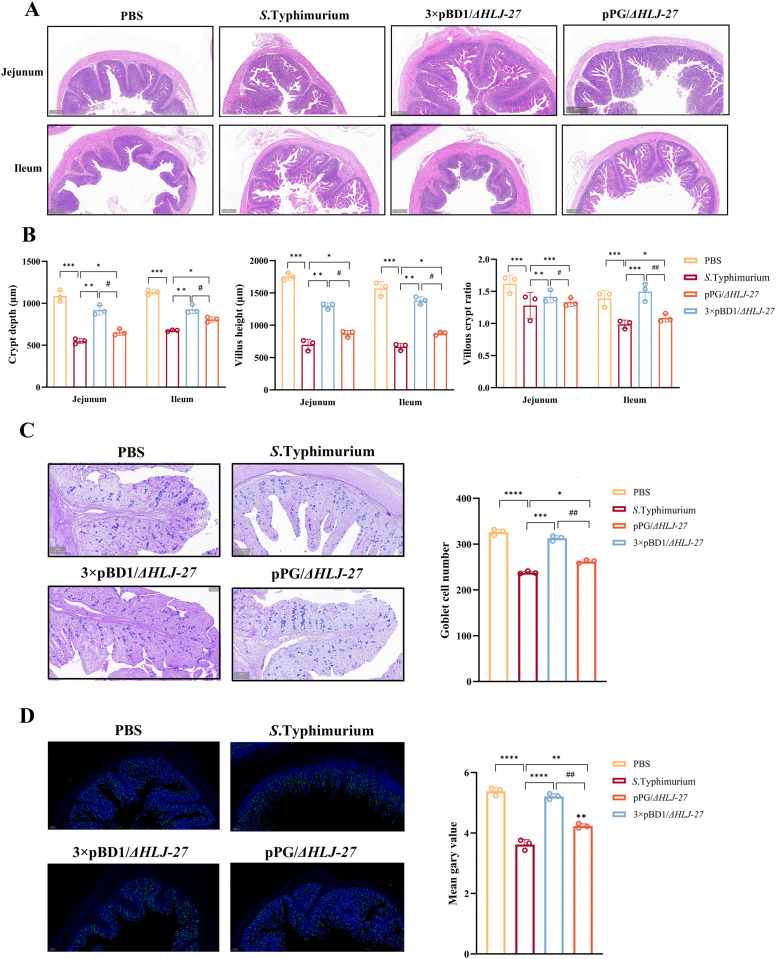
3 × pBD1/*ΔHLJ-27* protects intestinal morphology and mucus barrier during *S.* Typhimurium infection in piglets. **A**. Representative images of the jejunum and ileum stained with H&E (scale bar, 500 µm) Green arrows indicate intestinal villus shedding lesions. **B**. Measurement of the villi length, crypt depth and the villus-crypt ratio in the jejunum and ileum intestine. **C**. Representative images of the ileum stained with AB-PAS (scale bar, 100 µm). The -blue-purple goblet-shaped cells represent goblet cells. **D**. Immunofluorescence staining of the ileum with anti-MUC2 (in green; cell nuclei in blue, scale bar, 100 µm). Note: Data are presented as mean ± SD; n = 3 per group. ^*^P < 0.05, ^**^P < 0.01, ^***^P < 0.001 vs *S.* Typhimurium, ns, *P* > 0.05. ^#^*P* < 0.05, 0.01 < ^##^*P* < 0.05, ^###^*P* < 0.01, 3 × pBD1/*ΔHLJ-27* vs pPG/*ΔHLJ-27*; ns, *P* > 0.05.

Morphological analyses revealed that *S.* Typhimurium infection led to inflammatory infiltration and intestinal injury in the jejunum and ileum tissues of piglets, compared with the *S.* Typhimurium-infected group. Oral administration of 3 × pBD1/*ΔHLJ-27* to neonatal piglets attenuated *S.* Typhimurium-infected inflammatory infiltration and improved the structure of the intestinal mucosa (Fig 6A). However, the quantitative analysis showed that piglets treated with 3 × pBD1/*ΔHLJ-27* and pPG-PPT/*ΔHLJ-27* experienced a significant enhancement in villus height and villus-to-crypt ratio in the middle jejunum and distal ileum compared to the *S.* Typhimurium-infected group (Fig 6B). Goblet cell distribution, as visualized by AB-PAS staining, revealed a substantial reduction in goblet cells in the *S.* Typhimurium-infected group, while the 3 × pBD1/*ΔHLJ-27* group demonstrated significant restoration of goblet cell numbers (Fig 6C). Subsequent examinations demonstrated a significant reduction in fluorescence intensity in the *S.* Typhimurium-infected group in comparison to the PBS group. Conversely, the 3 × pBD1/*ΔHLJ-27* group exhibited a substantial enhancement in MUC2 intensity (Fig 6D).

**Fig 7:**
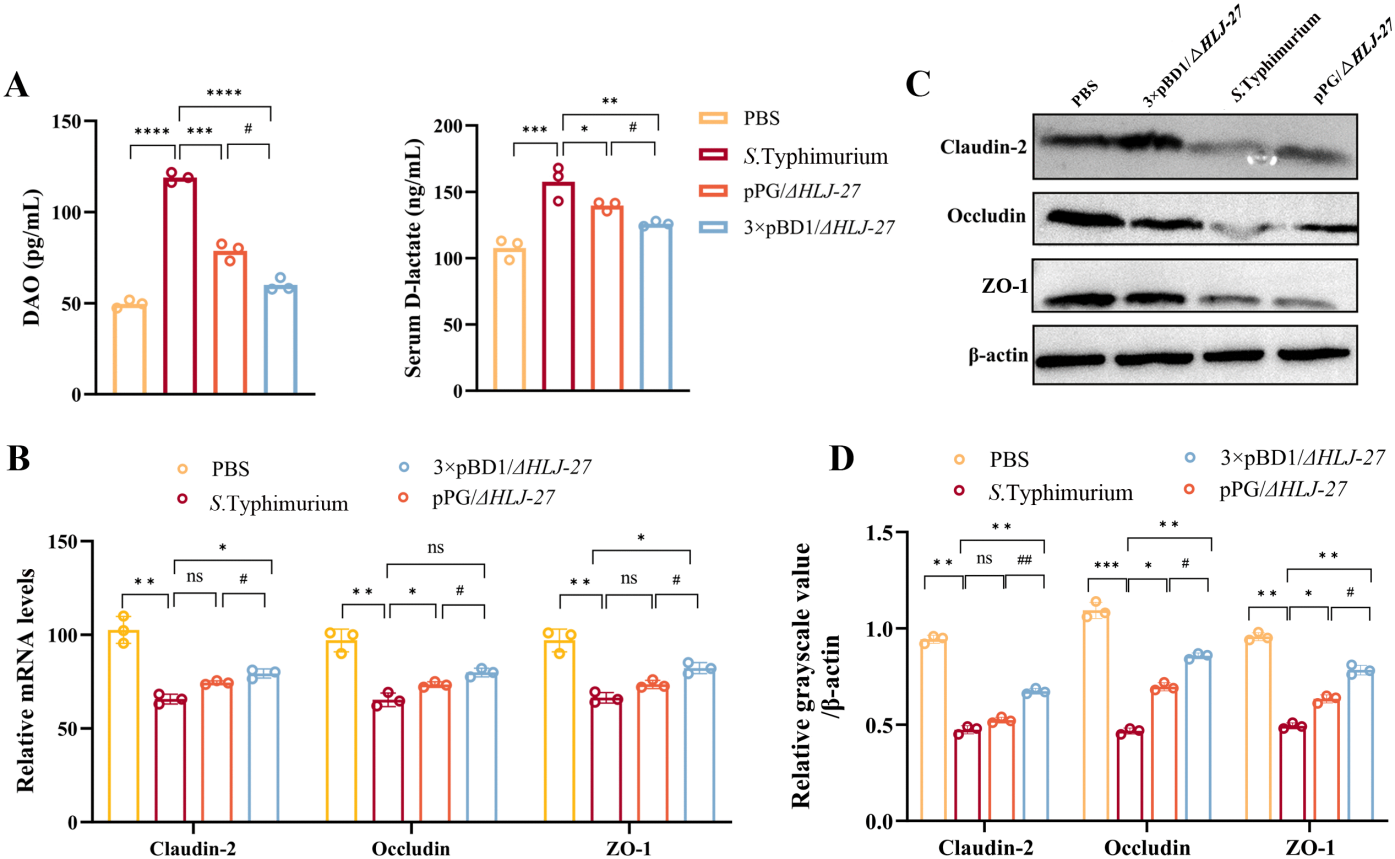
3 × pBD1/*ΔHLJ-27* restores intestinal barrier integrity by regulating tight junction proteins following Salmonella infection in piglets. **A**. The serum DAO and D-lactic acid levels of piglets. **B**. Real-time PCR analysis of tight junction protein expression in ileum of piglets. **C, D**. Western blot analysis of tight junction protein expression in ileum epithelium of piglets. Note: Data are presented as mean ± SD; n = 3 per group. ^*^P < 0.05, ^**^P < 0.01, ^***^P < 0.001 vs *S.* Typhimurium; ^#^*P* < 0.05, 0.01 < ^##^*P* < 0.05, ^###^*P* < 0.01, 3 × pBD1/*ΔHLJ-27* vs pPG/*ΔHLJ-27*; ns, *P* > 0.05.

Furthermore, concentrations of D-lactic acid and DAO were significantly reduced in the 3 × pBD1/*ΔHLJ-27* and pPG/*ΔHLJ-27* groups relative to the *S.* Typhimurium-infected group (Fig 7A), indicating an improvement in intestinal barrier integrity. At the molecular level, the expression of mRNA for the tight junction proteins ZO-1, Claudin-2, and Occludin was significantly upregulated in both the 3 × pBD1/*ΔHLJ-27* and pPG-PPT/*ΔHLJ-27* groups compared to the *S.* Typhimurium-infected group, with the 3 × pBD1/*ΔHLJ-27* group exhibiting the highest expression levels (Fig 7B). Western blot analysis indicated an upregulation of tight junction proteins (ZO-1, Occludin, and Claudin-2), in the recombinant strain relative to *S.* Typhimurium-infected group (Fig 7C and 7D).

### Mechanistic insights into systemic immune activation by recombinant strain 3 × pBD1/*ΔHLJ-27* in piglets

**Fig 8:**
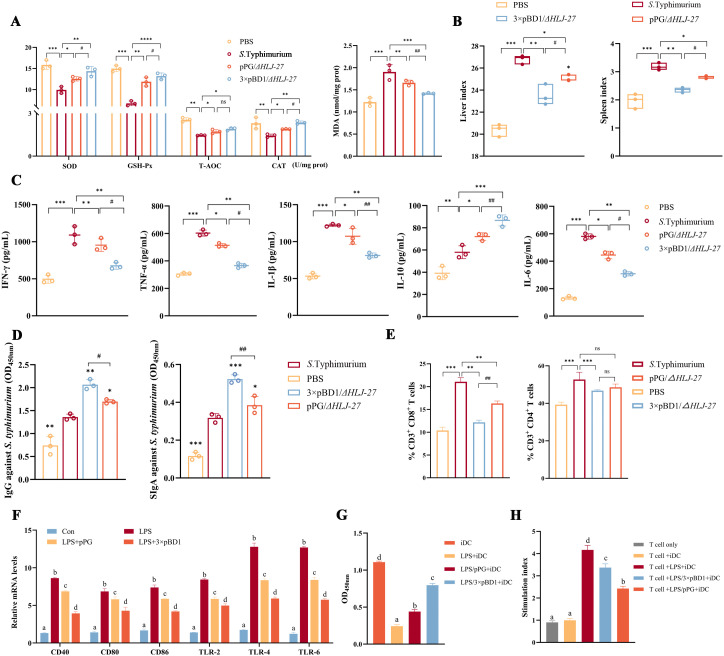
Recombinant strain 3 × pBD1/*ΔHLJ-27* confers systemic protection against *S.* Typhimurium infection by orchestrating integrated immune and antioxidant responses in piglets. **A**. Changes in serum antioxidant indices (SOD, GSH-Px, T-AOC, CAT activities and MDA content) in piglets. **B.** Determination of organ index in piglets infected with ***S.*** Typhimurium*.* **C.** Determination of inflammatory secretion level of piglets. **D**. Determination of anti-*S.* Typhimurium specific antibody responses of piglets. **E.** Percentage of splenic CD4^+^ and CD8^+^ T cells determined by flow cytometry. **F.** Effect of 3 × pBD1 expressed by 3 × pBD1/*ΔHLJ-27* on LPS-stimulated surface molecules and Toll-like receptors of MoDCs. **G.** Effect of 3 × pBD1 expressed by 3 × pBD1/*ΔHLJ-27* on LPS-stimulated phagocytosis of MoDCs. **H.** Effect of 3 × pBD1 expressed by 3 × pBD1/*ΔHLJ-27* on LPS-stimulated MoDCs to mediate CD4^+^ T cell proliferation. Note: Data are presented as mean ± SD; n = 3 per group. ^*^P < 0.05, ^**^P < 0.01, ^***^P < 0.001 vs ***S.*** Typhimurium; ^#^*P* < 0.05, 0.01 < ^##^*P* < 0.05, ^###^*P* < 0.01, 3 × pBD1/*ΔHLJ-27* vs pPG/*ΔHLJ-27*; ns, *P* > 0.05. Different letters (a vs. b, a vs. c, a vs. d, b vs. c, b vs. d, c vs. d) denote statistically significant differences (*P* < 0.05).

The enzymatic activities of SOD, T-AOC, CAT, and GSH-Px in the small intestine were significantly decreased following *S.* Typhimurium infection; however, the 3 × pBD1/*ΔHLJ-27* group demonstrated reduced MDA levels and a restoration of antioxidative enzyme activities (Fig 8A). The strain significantly reduced the infection-induced pathological enlargement of immune organs, as evidenced by the notably lower hepatosplenic indices in the 3 × pBD1/*ΔHLJ-27* group compared to the infected group (Fig 8B). In the serum of piglets infected to *S.* Typhimurium, there was a significant elevation in pro-inflammatory cytokines, including IL-1β, IL-12, IL-6, and TNF-α, while IL-10 levels were notably decreased. Treatment with 3 × pBD1/*ΔHLJ-27* resulted in a significant reduction in the production of pro-inflammatory cytokines and an increase in IL-10 levels. Importantly, the effects observed in the 3 × pBD1/*ΔHLJ-27* group surpassed those in the pPG-PPT/*ΔHLJ-27* group (Fig 8C).

Infection with *S.* Typhimurium led to a marked increase in serum IgG and intestinal SIgA levels. Vaccination via oral administration of 3 × pBD1/*ΔHLJ-27* resulted in notably elevated titers of antigen-specific IgG and SIgA compared to the infected group (Fig 8D). Flow cytometric analysis of mesenteric lymph nodes showed that *S.* Typhimurium infection significantly increased the proportions of CD4⁺ and CD8 ⁺ T cells compared with the PBS group (*P* < 0.001). Pretreatment with pPG-PPT/*ΔHLJ-27* and the recombinant strain 3 × pBD1/*ΔHLJ-27* significantly reduced CD8 ⁺ T cell levels, with a more pronounced effect observed in the recombinant strain group (*P* < 0.01). The proportion of CD4^+^T-cell was significantly different between the recombinant strain-treated group and the infected group (*P* < 0.05), whereas no significant difference was observed between the recombinant strain and the parental strain groups (Fig 8E). The proportion of CD4 ⁺ T cells increased in all treatment groups compared with the control, with the highest level observed in the *S.* Typhimurium group. The 3 × pBD1/*ΔHLJ-27* group showed a reduced level, suggesting a modulatory effect on T cell activation. (S3 Fig).

Further exploration of the effects of 3 × pBD1 on the development and performance of MoDCs demonstrated that 3 × pBD1 treatment notably decreased the excessive expression of surface molecules and TLRs triggered by LPS (Fig 8F). In comparison, the group treated with the empty vector also decreased the overexpression of MoDC surface markers, albeit to a lesser extent than the impact observed with 3 × pBD1. These findings imply that 3 × pBD1 might regulate DCs activation and antigen recognition functions. The phagocytic capacity of MoDCs was evaluated using the neutral red uptake assay (Fig 8G). The results indicated that LPS-stimulated MoDCs displayed diminished phagocytic activity compared to their unstimulated counterparts. In contrast, MoDCs co-treated with LPS and 3 × pBD1 demonstrated a significant enhancement in phagocytosis, while the LPS + pPG group showed only a modest increase in phagocytic activity. These findings suggest that 3 × pBD1 enhances the phagocytic capacity of MoDCs, thereby contributing to the host’s immune defense. The capacity of 3 × pBD1/*ΔHLJ-27* to promote T cell proliferation through LPS-stimulated MoDCs was assessed via the CCK-8 assay (Fig 8H). Co-culturing T cells with LPS-treated MoDCs notably boosted T cell proliferation. Nevertheless, both 3 × pBD1 and the empty vector protein hindered LPS-induced T cell proliferation, with 3 × pBD1 exhibiting a more potent inhibitory influence. These outcomes indicate that 3 × pBD1 regulates T cell responses, potentially impacting adaptive immune reactions. In summary, these results illustrate that 3 × pBD1/*ΔHLJ-27* not only diminishes bacterial colonization and boosts immune responses but also plays a crucial role in modulating both innate and adaptive immune pathways in reaction to *S.* Typhimurium infection.

### Integrated analysis of gut microbiota and host transcriptome reveals mechanisms underlying the protective effects of the recombinant strain

The microbiota composition and diversity of cecal contents on day 7 post-*S.* Typhimurium infection were evaluated through deep sequencing of the V3-V4 region of the 16S rRNA genes. Rank abundance curves analysis confirmed sufficient sequencing depth across all groups (PBS, ST, pPG, and pBD1), indicating adequate coverage of microbial diversity. Notably, species richness was significantly higher in the pBD1 group (S2A Fig). Principal coordinates analysis (PCoA) based on weighted UniFrac distances revealed that pPG and pBD1 treatments markedly altered the gut microbial community structure, leading to distinct clustering patterns (S2B Fig). Receiver operating characteristic (ROC) analysis further demonstrated strong discriminative power among groups, with area under the curve (AUC) values approaching 1, indicating clear differences in microbial profiles (S2C Fig).

**Fig 9:**
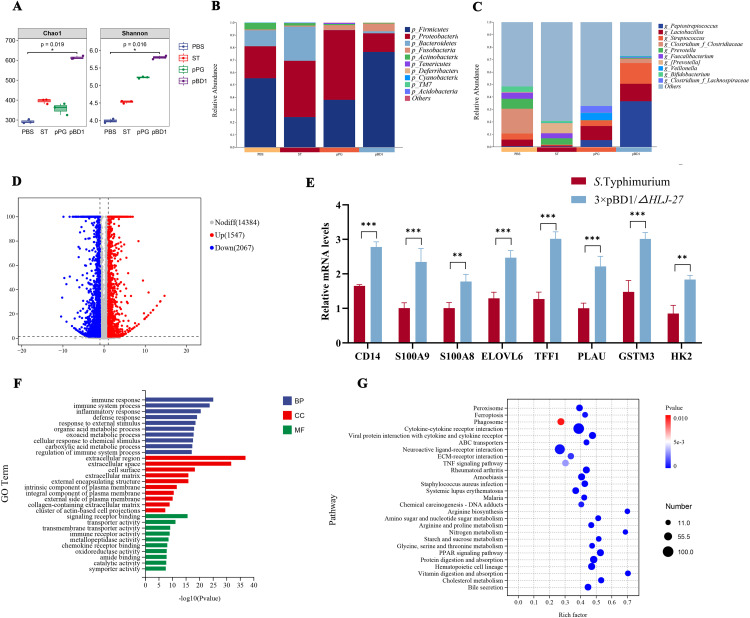
Effects of 3 × pBD1/*ΔHLJ-27* on intestinal microflora and ileum tissue in mice. **A**. Analysis of α-diversity index of community diversity (Shannon). and richness (Chao). Relative abundance of gut bacterial phylum (**B**) and family (**C**) among groups. **D**. up-and downregulated GO terms in 3 × pBD1/*ΔHLJ-27* and ***S.*** Typhimurium group*.* **E**. Validation of RNA-seq by qRT-PCR. **F**. GO enrichment analysis of differentially expressed genes. **G**. KEGG enrichment analysis of differentially expressed genes. Note: Data are presented as mean ± SD; n = 3 per group. ^*^P < 0.05, ^**^P < 0.01, ^***^P < 0.001; ns, *P* > 0.05.

Additional α-diversity analysis, employing the Chao1 and Shannon indices, demonstrated that the 3 × pBD1/*ΔHLJ-27* and pPG-PPT/*ΔHLJ-27* treatments significantly increased gut microbiota richness and diversity in comparison to the ST group (Fig 9A). At the phylum level, *Bacteroidetes*, *Firmicutes*, *Proteobacteria*, and *Fusobacteria* predominated in the cecal microbiota (Fig 9B). Compared to the infection group, oral administration of 3 × pBD1/*ΔHLJ-27* and pPG-PPT/*ΔHLJ-27* increased the relative abundance of *Firmicutes* while decreasing that of *Bacteroidetes*, *Proteobacteria*, and *Fusobacteria*. Notably, 3 × pBD1/*ΔHLJ-27* had a more pronounced effect than pPG-PPT/*ΔHLJ-27*. At the genus level, treatments with pPG and pBD1 resulted in a significant increase in *Lactobacillus* abundance, while concurrently inhibiting *Streptococcus* and *Prevotella*. The pBD1 treatment demonstrated a stronger effect in promoting beneficial bacteria. In contrast, pPG treatment enriched *Peptostreptococcus*, a facultative pathogen associated with opportunistic infections in immunocompromised individuals (Fig 9C). Analysis of dominant strains across cohorts indicated that *Faecalibacterium* and *Lactobacillus* were most prevalent in the model group, suggesting that these two treatments significantly influenced microbiota composition. Venn diagram analysis of shared microbial abundance further illustrated that, relative to the ST group, the pBD1 group exhibited varying degrees of microbial recovery (S2D Fig). LEfSe (LDA Effect Size) analysis identified *Mollicutes* and *Tenericutes* as prominent classes in the 3 × pBD1/*ΔHLJ-27* group, with *Mollicutes* classified as pathogenic organisms in animals (S2E Fig).

The pBD1 group demonstrated enhanced activity in antioxidant defense and nucleotide synthesis pathways, implying an improved capacity to manage oxidative stress. In contrast, the ST group displayed diminished activity in metabolic pathways such as peptidoglycan synthesis and folate metabolism, potentially reflecting inhibited cellular growth or metabolic function. Collectively, these findings illustrate that the 3 × pBD1/*ΔHLJ-27* treatment significantly modifies gut microbiota composition and functional pathways, offering potential advantages for host metabolism and immune defense (S2F and S2G Fig).

To further investigate whether these microbiota-associated functional alterations are linked to host responses, transcriptomic analysis was subsequently performed. A total of 1547 upregulated and 2067 downregulated DEGs were identified in the *S.* Typhimurium infection vs. 3 × pBD1/*ΔHLJ-27* group comparison (Fig 9D). Several genes related to immunity, gut barrier integrity, and metabolism were significantly upregulated in the 3 × pBD1/*ΔHLJ-27*-treated group compared with the *S.* Typhimurium-infected group. Specifically, immune-related genes such as CD14 (cluster of differentiation 14), S100A8 (S100 calcium-binding protein A8), and S100A9 (S100 calcium-binding protein A9) were markedly increased. Genes associated with gut barrier integrity, including TFF1 (trefoil factor 1) and PLAU (urokinase-type plasminogen activator), were also upregulated. In addition, metabolism-related genes, such as ELOVL6 (elongation of very long-chain fatty acids protein 6), HK2 (hexokinase 2), and GSTM3 (glutathione S-transferase mu 3), showed significant upregulation. Furthermore, qPCR validation confirmed that the expression patterns of these genes were consistent with the transcriptomic analysis results (Fig 9E).

GO enrichment analysis revealed that differentially expressed genes were significantly enriched in biological processes related to immune response, inflammatory response, and defense response. In terms of cellular components, these genes were mainly associated with the extracellular region, extracellular matrix, and plasma membrane. Molecular function analysis further indicated enrichment in signaling receptor binding, immune receptor activity, and oxidoreductase activity (Fig 9F). KEGG pathway analysis showed that the DEGs were primarily involved in immune-related pathways, including cytokine-cytokine receptor interaction, TNF signaling pathway, and phagosome. In addition, pathways associated with oxidative stress (peroxisome, ferroptosis), extracellular matrix interaction (ECM-receptor interaction), and metabolic processes (amino acid metabolism, PPAR signaling pathway) were significantly enriched (Fig 9G).

## Discussion

The intestine functions as a key barrier against pathogen invasion while also serving as a central site for nutrient absorption and immune regulation [34]. *L. paracasei* is a widely used probiotic species in humans [35]. This study provides a comprehensive evaluation of intestinal mucosal immune responses associated with the pBD1-expressing *L. paracasei* strain, as well as an overall assessment of its biological activity and potential for anti-infective applications.

In previous laboratory work, the recombinant strain 3 × pBD1/*ΔHLJ-27* was validated at the protein level by Western blot, confirming stable pBD1 expression in both culture supernatant and bacterial lysates without affecting bacterial growth or physiological characteristics. A key limitation of this study is the absence of direct *in vivo* validation for pBD1 production by the engineered bacteria, and our observations only support correlative associations rather than confirmed causal mechanisms underlying host protection. Building on these findings, the colonization ability of 3 × pBD1/*ΔHLJ-27* in mice and piglets was assessed to determine its potential for sustained intestinal persistence and long-term immunomodulatory effects. Plate counting and real-time PCR results showed that the recombinant strain primarily colonized the jejunum and ileum of mice and piglets. Since intact intestinal morphology supports intestinal barrier function and nutrient absorption [36], such structural maintenance may contribute to improved growth performance. In this study, 3 × pBD1/*ΔHLJ-27* increased body weight in both mice and piglets. Furthermore, H&E staining demonstrated that the recombinant strain preserved ileal villus architecture and crypt integrity. Immune organs are core components regulating host immune homeostasis [37,38]. 3 × pBD1/*ΔHLJ-27* potentially promoted the development of the thymus, spleen and mesenteric lymph nodes, alongside increased CD4 ⁺ T cell abundance in mesenteric lymph nodes. Specifically, the levels of serum IgG and intestinal mucosal SIgA were significantly elevated in mice and piglets treated with the 3 × pBD1/*ΔHLJ-27*. Wild-type *L. paracasei* exerts immunomodulatory effects via gut microbiota regulation [39], and pBD1 expression has the potential to work in concert with these intrinsic probiotic functions. However, the relative contribution of pBD1 expression versus intrinsic probiotic properties requires further investigation.

*S.* Typhimurium, a critical zoonotic pathogen, severely threatens animal health and food safety. In our mouse and piglet infection models, 3 × pBD1/*ΔHLJ-27* intervention improved host survival and alleviated disease severity, indicating protective potential against infection. *S.* Typhimurium disrupts intestinal barrier integrity by targeting epithelial junctions, which makes it more invasive in the host. Han et al. [40] showed that the pBD2 peptide made in the lab can increase the levels of mRNA for certain proteins that help keep the intestinal barrier strong, which can help reduce damage from inflammation in the colon. In this context, the protective effects of 3 × pBD1/*ΔHLJ-27* are reflected in the preservation of mucosal architecture and the restoration of goblet cell populations, which may be partially associated with maintenance of epithelial integrity mediated by the combined effects of *L. paracasei* and pBD1. Given the essential role of goblet cells in mucin production, their preservation likely supports the mucosal barrier and enhances resistance to pathogen invasion [41–43]. Consistent with reduced goblet cell populations, *S.* Typhimurium infection suppressed intestinal MUC2 expression, a key component of the mucosal barrier [44]. The recovery of MUC2 induced by 3 × pBD1/*ΔHLJ-27* may be linked to maintained mucin production and barrier function potentially driven by the combined effects of *L. paracasei* and pBD1 expression.

The function of the intestinal barrier is related to many factors, including D-lactate, DAO in serum, and intestinal epithelial TJPs [45,46]. In this study, *S.* Typhimurium infection impaired barrier function, as reflected by increased serum D-lactate and DAO levels and reduced expression of tight junction proteins. In contrast, treatment with 3 × pBD1/*ΔHLJ-27* alleviated these changes, indicating preservation of epithelial integrity. Given that disruption of tight junctions facilitates bacterial translocation to systemic organs, these findings suggest that the recombinant strain limits pathogen dissemination by maintaining barrier function [47–50]. Oral administration of 3 × pBD1/*ΔHLJ-27* alleviated intestinal damage and reduced systemic bacterial burden. These findings suggest that the recombinant strain may limit *S.* Typhimurium dissemination and protect immune organs, likely through preservation of intestinal barrier integrity. In addition, lower bacterial loads in the jejunum and ileum at 7, 14, and 21 days suggest reduced intestinal persistence. Together, these findings support that the engineered strain exerts protective effects not only by restricting extraintestinal spread, but also by suppressing intestinal colonization.

The intestinal barrier is shaped by dynamic interactions among microbiota, epithelial cells, and immune components [51]. Our results show that treatment with 3 × pBD1/*ΔHLJ-27* increased microbial richness following *S.* Typhimurium infection. This preservation of microbial complexity may contribute to enhanced resistance to pathogen invasion and support intestinal homeostasis. These changes featured enriched beneficial genera such as *Lactobacillus* and *Faecalibacterium*, and decreased potential pathogens. These microbial shifts may help improve intestinal barrier and immune function, though direct causal relationships remain to be verified. To further explore whether these microbiota changes are associated with host responses, transcriptomic analysis was performed. Transcriptomic analysis further supported the protective effects of 3 × pBD1/*ΔHLJ-27* at the molecular level. Differentially expressed genes were predominantly enriched in pathways related to immune regulation, barrier function, and metabolism, consistent with the observed improvements in intestinal integrity and host defense. Notably, the upregulation of genes associated with innate immunity and epithelial repair suggests enhanced host responsiveness to infection. In addition, enrichment of metabolic and oxidative stress-related pathways indicates a broader role in maintaining intestinal homeostasis. Together, these findings provide mechanistic insight into how the recombinant strain coordinates immune, barrier, and metabolic responses to counteract *S.* Typhimurium infection.

Oxidative stress has been demonstrated to leads to intestinal damage, resulting from the overproduction of MDA and the decrease in antioxidant defense [52,53]. In the present study, we observed that *S.* Typhimurium challenge caused intestinal oxidative injury, indicated by an increase in the levels of MDA and a decrease in the activities of SOD, GSH-Px, CAT and T-AOC. However, oral administration of 3 × pBD1/*ΔHLJ-27* to neonatal piglets alleviated these indicators, indicating 3 × pBD1/*ΔHLJ-27* could improve the antioxidant capacity of newborn piglets.

Dendritic cells (DCs) play a central role in linking innate and adaptive immunity by regulating T cell responses. Defensins have been reported to modulate DCs maturation and function [54–56]. In this study, 3 × pBD1 enhanced the expression of co-stimulatory molecules on DCs, indicating promoted maturation. Notably, 3 × pBD1 also suppressed LPS-induced CD4 ⁺ T cell activation and proliferation mediated by MoDCs, suggesting a potential mechanism for its anti-inflammatory effects. Although the use of purified pBD1 isolates the peptide’s specific immunomodulatory effects, this *in vitro* model does not fully replicate the complex *in vivo* environment where the *L. paracasei* vector continuously secretes the peptide. Therefore, while our *in vitro* findings highlight the intrinsic potential of pBD1, the observed *in vivo* immune modulation likely results from a synergistic interaction between the probiotic backbone and the expressed defensin.

Defensins have been reported to modulate CD4 ⁺ T cell responses, potentially through pathways involving dendritic cell activation and antigen presentation [57]. Consistent with this, treatment with 3 × pBD1/*ΔHLJ-27* increased CD4 ⁺ T cell levels, suggesting enhanced cellular immunity against *S.* Typhimurium. In addition, defensins are associated with Th1-biased responses and antibody production [58]. In our study, this recombinant strain correlated with elevated systemic IgG and mucosal SIgA, which may collectively strengthen host defense against pathogen invasion [59]. In this study, infection induced a marked inflammatory response, whereas treatment with 3 × pBD1/*ΔHLJ-27* reduced pro-inflammatory cytokines and increased IL-10 levels. IL-6 is linked to immune activation and chronic inflammation [60], while IL-10 suppresses inflammation and aids tissue repair [61]. 3 × pBD1/*ΔHLJ-27* may alleviate *S.* Typhimurium triggered inflammation possibly by balancing Th1 and Th2 related immune responses.

Probiotic strategies are increasingly explored as antimicrobial alternatives. For instance, Bacillus-based interventions against *E. coli* typically rely on multi-strain formulations to broadly modulate the gut microbiota [28]. In contrast, our engineered *Lactobacillus* approach against *S.* Typhimurium achieves targeted protection by directly delivering the pBD1 peptide to reinforce epithelial barrier function and local immunity. However, the gastrointestinal environment imposes harsh physiological stresses. Compared with multi-strain probiotic mixtures with better tolerance to gastric acid and bile salts [24], single-strain systems may face ecological limitations. Nevertheless, our results suggest that 3 × pBD1/*ΔHLJ-27* can compensate for this reduced ecological complexity by providing strong, localized antimicrobial efficacy. Accordingly, this engineered single strain probiotic may offer a complementary alternative to multi-strain probiotic mixtures for intestinal pathogen control.

This study validated the recombinant strain 3 × pBD1/*ΔHLJ-27* in a cross-species *S.* Typhimurium infection model. Consistent with previous reports that lactic acid bacteria can modulate both innate and adaptive immunity [62]. Our findings suggest that 3 × pBD1/*ΔHLJ-27* reduces morbidity and mortality following *S.* Typhimurium infection by enhancing host defense, likely through the antimicrobial and immunomodulatory activities of pBD1. The observed protection appears to be associated mainly with modulation of host responses and competitive exclusion, rather than with direct interference in pathogen-specific virulence processes, which were not assessed in the present study. However, because other porcine pathogens were not evaluated and intestinal bacterial burden was not directly quantified, the broad-spectrum efficacy and local antimicrobial mechanisms of this recombinant strain remain to be further clarified. Future studies are therefore needed to address these questions and to better define the host-pathogen interactions underlying its protective effects.

## Materials and methods

### Ethics statement

The procedures for sampling and processing piglets or mouse were reviewed and approved by the Animal Ethics Committee of the School of Northeast Agricultural University. The Animal Ethics Committee approval number was NEAUEC 20220318. All animals were performed in accordance with animal ethics guidelines and approved protocols.

### Bacterial strain

pPG-T7g10-PPT/*ΔHLJ-27* (pPG/*ΔHLJ-27*) preserved in our laboratory strain repository. pPG-TSP-3 × pBD1/*ΔHLJ-27* (3 × pBD1/*ΔHLJ-27*) developed and maintained by our research team. The alanine racemase-deficient *Lactobacillus paracasei* strain (*ΔAlr-HLJ-27*), developed and maintained in our laboratory, is an auxotrophic mutant necessitating D-alanine supplementation (200 μg/mL) for optimal development. *Salmonella* Typhimurium CMCC 50115 (*S.* Typhimurium) acquired from the China Institute of Veterinary Drug Control (IVDC), the official veterinary drug assessment facility under the Ministry of Agriculture and Rural Affairs.

### Effect of oral administration of the recombinant strain 3 × pBD1/*ΔHLJ-27* on the growth performance of mice

#### Animals and experimental design.

70 specific pathogen-free (SPF) BALB/c mice, each 8 weeks old, were sourced from Liaoning Changsheng Biotechnology Co. To investigate the effects of the recombinant strain 3 × pBD1/*ΔHLJ-27* on the growth performance of mice. Each group was given different treatments as follows: (1) oral administration of 200 μL PBS to mice from day 1 to day 7. (2) oral administration of 3 × pBD1/*ΔHLJ-27* (1.0 × 10^10^ CFU/ mL dissolved in 200 μL of PBS) to mice from day 1 to day 7. S1A Fig indicate the oral administration processes for mice.

Mice in the control group were orally administered PBS, which served as a procedural control to account for potential effects associated with the administration procedure. PBS is widely used as a vehicle control in studies involving oral delivery of probiotics or bacterial strains.

#### Quantitative analysis of recombinant strain colonization by plate counting and RT-qPCR in mice.

The intestinal colonization of the recombinant strain 3 × pBD1/*ΔHLJ-27* in mice was evaluated using a standardized plate counting technique. After 7 consecutive days of oral dosing, intestinal mucus samples were obtained from the duodenum, jejunum, ileum, cecum, and colon on days 7, 14, 21, and 28. Samples were serially diluted in PBS and inoculated onto MRS agar enriched with 5 µg/mL chloramphenicol (Cm) and 200 μg/mL D-alanine. Following 24–30 hours of static incubation at 37°C, colony counts were conducted to assess the spatiotemporal colonization patterns across intestinal segments.

In parallel, the same intestinal mucus samples were subjected to molecular quantification. A standard curve was created for absolute quantification utilizing the *Cm* gene (encoded in pPG-PPT/*ΔHLJ-27*) as a reference point. The 19T-qCm plasmid (1 × 10^9^ copies/μL) underwent serial 10-fold dilutions, and the log10 plasmid copy numbers were plotted against Ct values to create the calibration curve. The total bacterial DNA collected from intestinal mucus was examined using real-time PCR, with recombinant bacterial loads measured relative to the standard curve.

#### Measurement of body weight.

After a 7-day continuous oral administration regimen of either the recombinant strain 3 × pBD1/*ΔHLJ-27* or an equivalent volume of PBS (control), the mice underwent systematic body weight assessments and health status evaluations. Weight measures were conducted at uniform intervals (days 0, 3, 7, 10, 14, 18, 21, 24, and 28 post-administration) under controlled conditions, with all assessments occurring at the same time of day (± 1 hour) to reduce circadian fluctuations.

#### Histological analysis.

Intestinal segments were collected aseptically, washed with PBS, and preserved in 4% paraformaldehyde for 48 hours. After paraffin embedding and sectioning at 4 μm, hematoxylin and eosin (H&E) staining was conducted to assess villus morphology [63]. Villus height, crypt depth, and their ratio (VH/CD) were quantified using ImagePro Plus 6.0, with a minimum of 10 intact villus-crypt units evaluated in each sample to determine mucosal integrity.

#### Determination of immune organ indices.

On day 28 post-treatment, the liver, spleen, thymus, and mesenteric lymph nodes were meticulously removed and weighed. The indices of immunological organs were computed using standardized formulas: organ index = organ weight (g)/body weight (g).This standardized metric facilitates the comparative assessment of immune organ development among animals of varying sizes.

#### Determination of immunoglobulin levels.

This assessment quantified serum IgG antibody levels and SIgA concentrations in intestinal mucus, as well as serum cytokine profiles (Jiangsu Meimian Industrial Co., Ltd., Shanghai, China), following the manufacturers’ recommended protocols.

### Protective effects of oral administration of the recombinant strain against *S.* Typhimurium infection in mice

#### Animals and experimental design.

Study on the anti-infection effect of oral administration of 3 × pBD1/*ΔHLJ-27* in mice. Each group was given different treatments as follows: (1) Control (PBS) group (ten mice from control treatment and oral administration of 200 μL sterile PBS only); (2) ST (*S.* Typhimurium) group (ten mice from control treatment and oral challenge with 5.0 × 10^8^ CFU/ mL dissolved in 200 μL PBS); (3) ST + pPG-PPT/*ΔHLJ-27* (pPG-PPT/*ΔHLJ-27*) group (ten mice from pPG-PPT/*ΔHLJ-27* treatment and oral challenge with 5.0 × 10^8^ CFU/mL dissolved in 200 μL PBS); (4) ST + 3 × pBD1/*ΔHLJ-27* (3 × pBD1/*ΔHLJ-27*) group (ten mice from 3 × pBD1/*ΔHLJ-27* treatment and oral challenge with 5.0 × 10^8^ CFU/ mL dissolved in 200 μL PBS). Fig 3A indicate the oral administration processes for mice.

#### Disease progression and survival analysis in mice.

After 7 days oral inoculation with recombinant strain, the animals were infected by *S.* Typhimurium on day 8. Clinical symptoms, encompassing physical state, gastrointestinal manifestations, and neurological function, were assessed daily (refer to Tables 1 and 2), whereas mortality was evaluated every 12 hours (humane endpoint: ≥ 20% weight reduction). The disease activity index (DAI) and survival rates were evaluated by Kaplan-Meier analysis with a log-rank test conducted by blinded observers to objectively measure vaccination efficacy [12].

**Table 1:** Criteria for assessment of Disease Activity Index (DAI).

DAI parameters	Severity Level	Score
Weight loss	None/Normal	0
	1-5%	1
	5-10%	2
	10-20%	3
	20-30%	4
	> 30%	5
Stool Consistency	Normal	0
	Loose stool/mild diarrhea	2
	Watery diarrhea	4
Intestinal Bleeding	No bleeding	0
	Occult blood	2
	Gross bleeding	4

**Table 2:** Post-challenge clinical symptom scores in piglets.

Observation Item	Grading Standard	Score
Mental Status	Normal	0
	Depression	1
	Lethargy with decreased appetite	3
	Prostration with moribund state	4
Diarrhea Severity	Normal	0
	Soft stool	1
	Soft stool with watery content	3
	Watery diarrhea	4

Normal stool consistency is rated between 1 and 2, however values of 2.5 or above signify diarrhea. Clinical illness is characterized by either: (1) a fecal score of 3 or higher, or (2) a rectal temperature outside the parameters of 101.5 to 103.5°F. Temperature readings were obtained with calibrated digital thermometers with an accuracy of ± 0.1°F.

#### Determination of immune organ indices.

Following *Salmonella* challenge, the liver and spleen of mice were collected and weighed, along with body weight. The liver and spleen indices were subsequently calculated for each group. Immune organ indices were determined as described above.

#### Bacterial translocation measurement.

Following the *S.* Typhimurium challenge, liver and spleen tissues from mice were aseptically harvested and homogenized in ice-cold PBS. The homogenate was filtered using a copper mesh to achieve a single-cell suspension. A 100 μL portion of the suspension was inoculated onto SS agar (selective for *S.* Typhimurium) and incubated at 37°C for 18–24 hours. Bacterial colonies were subsequently counted and identified, and the translocation rate (positive organs/total organs × 100%) was computed. Statistical analysis was conducted utilizing suitable software.

#### Intestinal morphology and barrier function analysis.

Histological analysis was performed as described above. Ileal tissues were collected after Salmonella challenge to evaluate intestinal damage.

The expression of tight junction proteins such as ZO-1, Claudin-2, and Occludin in ileum tissues was evaluated using RT-qPCR (S1 Table). ZO-1, Claudin-2, and Occludin. Total RNA was isolated from intestinal tissues with Trizol reagent, subsequently undergoing reverse transcription to produce cDNA. The relative expression levels were determined using the 2^-ΔΔCt^ technique, employing β-actin as the internal control.

#### Humoral immune response and cytokine analysis.

This assessment quantified serum IgG antibody levels, intestinal SIgA concentrations, and serum cytokine profiles — including IFN-γ, IL-1β, IL-6, IL-10, and TNF-α — using commercial ELISA kits, following the manufacturers’ instructions.

### Effect of oral administration of the recombinant strain 3 × pBD1/*ΔHLJ-27* on the growth performance of piglets

#### Animals and experimental design.

10 Neonatal piglets (Duroc × Landrace × Large White) were obtained from Shiji Biotechnology (Suihua) Pig Breeding Co., Ltd. To investigate the effects of the recombinant strain 3 × pBD1/*ΔHLJ-27* on the growth performance of piglets. Each group was given different treatments as follows: (1) oral administration of 2 mL PBS to piglets from day 1 to day 7. (2) oral administration of 3 × pBD1/*ΔHLJ-27* (1.0 × 10^10^ CFU/ mL dissolved in 2 mL of PBS) to piglets from day 1 to day 7. S1A Fig indicate the oral administration processes for piglets.

#### Quantitative analysis of recombinant strain colonization by plate counting and RT-qPCR in piglets.

After three consecutive days of oral administration of the recombinant strain, intestinal mucus samples were collected from the duodenum, jejunum, ileum, cecum, and colon of neonatal piglets by gentle scraping. The intestinal colonization of the recombinant strain in piglets was evaluated as described for mice.

#### Effects of the recombinant strain on growth performance and immune function in piglets.

For histological analysis, jejunal and ileal tissues from piglets were examined by H&E staining. Villus height and crypt depth were measured using ImagePro Plus 6.0, and the VH/CD was calculated. The expression levels of tight junction proteins (ZO-1, Occludin, and Claudin-2) in ileal tissues were evaluated using real-time PCR and Western blot analysis. The real-time PCR procedures were performed as described above. The intestinal tissue samples were rinsed with cold PBS and lysed using RIPA lysis buffer. Identical quantities of protein were submitted to SDS-PAGE and subsequently electrotransferred onto nitrocellulose membranes. The membranes were incubated in 5% nonfat milk for 2 hours at room temperature and subsequently treated with a monoclonal antibody specific to tight junction protein. Following three washing with PBST, the membranes were incubated with an HRP-conjugated goat anti-mouse antibody for one hour at ambient temperature. Following the washing procedure, proteins were identified utilizing an ECL kit.

Immune organ indices were determined as described above. Organ index = organ weight (g)/body weight (kg). The proportion of CD4 ⁺ T lymphocytes in mesenteric lymph nodes was analyzed using flow cytometry as described for mice. Serum total IgG and intestinal mucus SIgA levels were measured using ELISA following the same procedures as described above.

### Protective effects of oral administration of the recombinant strain against *S.* Typhimurium infection in piglets

#### Animals and experimental design.

Study on the anti-infection effect of oral administration of 3 × pBD1/*ΔHLJ-27* in mice and piglets. Each group was given different treatments as follows: (1) Control (PBS) group (five piglets from control treatment and oral administration of 10 mL sterile PBS only); (2) ST (*S.* Typhimurium) group (five piglets from control treatment and oral challenge with 5.0 × 10^8^ CFU/ mL dissolved in 2 mL PBS); (3) ST + pPG-PPT/*ΔHLJ-27* (pPG-PPT/*ΔHLJ-27*) group (five piglets from pPG-PPT/*ΔHLJ-27* treatment and oral challenge with 5.0 × 10^8^ CFU/ mL dissolved in 2 mL PBS); (4) ST + 3 × pBD1/*ΔHLJ-27* (3 × pBD1/*ΔHLJ-27*) group (five piglets from 3 × pBD1/*ΔHLJ-27* treatment and oral challenge with 5.0 × 10^8^ CFU/ mL dissolved in 2 mL PBS). Fig 5A indicate the oral administration processes for mice and piglets.

Clinical symptoms and survival rates were monitored following *S.* Typhimurium challenge to assess disease progression. Immune organ indices were determined as described above, with body weight expressed in kilograms.

#### Bacterial translocation measurement.

Bacterial translocation was assessed by culturing liver and spleen homogenates as described for mice. *S.* Typhimurium colonization kinetics were evaluated using a plate counting method. At designated time points post-infection (7, 14, and 21 days), intestinal contents (ileum and cecum) as well as liver and spleen tissues were aseptically collected. Samples were homogenized in sterile PBS, serially diluted, and plated onto selective SS agar. After incubation at 37°C for 18–24 h, bacterial colonies were counted, and the results were expressed as CFU per gram of tissue.

#### Intestinal morphology and barrier function analysis.

Histological analysis and tight junction protein expression in ileal tissues were evaluated using H&E staining, real-time PCR, and Western blot following the same procedures as described above. To check mucins and the goblet cells, the sections were stained with AB-PAS reagents [64].

Tissue sections were deparaffinized following the aforementioned protocol. For blocking, sections were incubated with 1% bovine serum albumin (BSA) in PBS for 1 hour at room temperature. Subsequently, sections were incubated overnight at 4°C with a primary anti-MUC2 antibody (diluted 1:100 in 1% BSA). After washing, slides were incubated with a secondary FITC-conjugated goat anti-rabbit antibody diluted 1:500 in PBST for 1 hour at room temperature. Sections were then washed sequentially with PBST and PBS, and mounted using DAPI. Images of tissue sections were acquired via fluorescence microscopy, and fluorescence intensity was quantified using ImageJ software [65].

The expression levels of tight junction proteins (ZO-1, Occludin, and Claudin-2) in ileal tissues were evaluated using real-time PCR and Western blot analysis. The diamine oxidase (DAO) and the D-lactic acid levels in the serum were measured using commercial ELISA kits, respectively, according to the manufacturer’s protocol.

#### Assessment of antioxidant capacity.

Ileum mucosa samples were homogenized in ice-cold PBS at a ratio of 1:4 (weight/volume), followed by centrifugation at 2500 × g for 10 min at 4°C to prepare the mucosal supernatant for assessing intestinal antioxidant levels. Intestinal mucosa antioxidant indices, such as total antioxidant capacity (T-AOC), superoxide dismutase (SOD), malondialdehyde (MDA), catalase (CAT), and glutathione peroxidase (GSH-Px), were quantified using commercially available assay kits (Nanjing Jiancheng Institute of Bioengineering, Jiangsu, China). The measurements were performed with a UV-visible (UV-VIS) spectrophotometer, strictly following the protocols provided by the kit manufacturers.

#### Flow cytometry.

Mesenteric lymph nodes were aseptically harvested and homogenized in 1 mL of full RPMI-1640 media utilizing a syringe plunger. The cell suspension was filtered using a 70-μm mesh, washed, and adjusted to a concentration of 1 × 10^6^ cells/mL in PBS. For labeling, 500 μL of cells were treated with 0.5 μL of PE-conjugated anti-mouse CD4 monoclonal antibody at 37°C for 30 minutes in the dark, subsequently washed, and analyzed using flow cytometry using BD FACSCalibur.

Peripheral blood mononuclear cells (PBMCs) were extracted from blood using Ficoll-Paque density gradient centrifugation. The interface layer cells were stained with anti-pig CD4-PE and CD3-FITC monoclonal antibody (37°C, 30 minutes, in the dark), using unstained cells as a negative reference. Following the PBS wash (500 rpm for 5 minutes), cells were resuspended in 500 μL PBS and subjected to flow cytometric analysis.

#### Microbiome analysis.

Microbial DNA was extracted from cecal contents of piglets using the E.Z.N.A. Stool DNA Kit (Omega Biotek, USA) according to the manufacturer’s instructions. Cecal samples were collected aseptically at the end of the experiment, immediately frozen, and stored at -80°C until analysis. A total of n = X biological replicates per group were subjected to 16S rRNA gene sequencing. The V3-V4 hypervariable region of the bacterial 16S rRNA gene was amplified using the primer pair 341F (5′-CCTACGGGNGGCWGCAG-3′) and 806R (5′-GGACTACHVGGGTATCTAAT-3′). PCR products were purified, quantified, and used for library construction. Sequencing was performed on the Illumina MiSeq platform (Illumina, San Diego, CA, USA) by Payseno Biotech (Shanghai, China). Raw reads were quality-filtered, merged, and chimeric sequences were removed prior to downstream analysis. High-quality reads were clustered into operational taxonomic units (OTUs) at 97% sequence similarity, and representative sequences were taxonomically assigned against the SILVA database. Alpha diversity was evaluated using the Chao1, Ace, Shannon, and Simpson indices, while beta diversity was analyzed by PCA and NMDS. Community overlap and differences among groups were further visualized using Venn diagrams and hierarchical clustering.

#### Transcriptome detection of ileum.

Total RNA was extracted, and mRNA was enriched using Oligo (dT) magnetic beads. The mRNA was fragmented into ~ 300 bp fragments and used as a template for first-strand cDNA synthesis with random hexamer primers, followed by second-strand cDNA synthesis. Libraries were constructed, PCR-amplified, and size-selected (~ 450 bp). Library quality was assessed using an Agilent Bioanalyzer, and concentrations were quantified. Indexed libraries were pooled and sequenced on an Illumina platform using paired-end (PE) mode. The raw sequencing data generated from the Illumina platform were processed as follows. Raw reads were filtered to remove adapters and low-quality sequences to obtain clean reads. Clean reads were aligned to the reference genome using HISAT2. Gene expression levels were quantified using featureCounts. Differentially expressed genes (DEGs) between groups were identified using DESeq2, with adjusted P-values < 0.05 as the significance threshold. GO and KEGG enrichment analyses were performed to explore the biological functions of DEGs.

#### Isolation of porcine peripheral blood mononuclear-derived dendritic cells (MoDCs) and detection of cell surface molecules and phagocytic ability.

The anterior vena cava blood of 4–7 weeks old piglets was collected with a vacuum blood collection tube. Immature porcine MoDCs were individually treated with 5 μg of the recombinant protein 3 × pBD1, an empty vector protein, or LPS (final concentration of 1 μg/mL). and incubated in a 37°C, 5% CO_2_ incubator for 12 hours. Immature porcine MoDCs cultured in complete medium containing an equal volume of RPMI-1640 basal medium were included as the negative control. After three washes with PBS, the cells were harvested using a cell scraper and subsequently transferred to Eppendorf tubes. The concentrations of cell surface molecules (CD40, CD80, and CD86) and Toll-like receptors (TLR2, TLR4, TLR6) were quantified (S2 Table).

Immature porcine MoDCs were seeded in 96-well plates. Once adherent, 5 μg of recombinant protein 3 × pBD1, empty vector control protein, or LPS (positive control) was individually added to the cultures. Following stimulation, cells were treated with mitomycin C at a final concentration of 25 μg/mL and incubated at 37°C for 1 hour. Treated porcine MoDCs were resuspended and washed with RPMI-1640 basal medium, followed by cell counting using a hemocytometer. PBMCs were resuspended in PBS, and the cell concentration was adjusted to 2 × 10^6^ cells/mL. The PBMCs were then resuspended in RPMI-1640 basal medium, with cell numbers quantified using a hemocytometer. Lymphocytes (100 μL) were added to porcine MoDCs subjected to different treatments at a stimulator cell:responder cell ratio of 1:10. Negative control wells contained immature porcine MoDCs alone or PBMCs alone, while blank control wells contained RPMI-1640 medium only. All wells were set up in triplicate with a final volume of 200 μL, and incubated in a 37°C, 5% CO_2_ incubator for 72 hours. 10 μL of CCK-8 reagent was added per well, after which the OD_450nm_ was measured using a microplate reader, and the calculatethe stimulation index (SI) of T cells according to the following formula: SI = (DC + T) OD_450nm_-(DC) OD_450nm_/(T) OD_450nm_-(medium) OD_450nm_.

A portion of MoDCs were placed in a 96-well plate at a density of 1 × 10^5^ cells, with 100μL of 0.1% neutral red added to each well, and then incubated at 37°C with 5% CO_2_ incubator. They were not phagocytosed and were discarded after 2 hours. Wash the neutral red with preheated PBS twice, then add 100 μL 1% SDS lysate to each well, let the cells belysed at room temperature for 2 hours, set 3 replicate wells in each group, and finally read on the microplate reader Take the OD_540nm_ value, the OD value is proportional to the phagocytic function of the cell.

### Statistical analysis

GraphPad Prism 10.1.2 software was used for statistical analysis and figure generation. All data were expressed as the mean ± SD. For experiments involving two independent variables, statistical significance was determined using two-way analysis of variance (ANOVA) followed by Sidak’s multiple comparisons test. Values with *P* < 0.05 were considered significantly different (^*^P < 0.05; ^**^P < 0.01; ^***^P < 0.001; ^****^P < 0.0001).

## Supporting information

S1 Fig 3 × pBD1/*ΔHLJ-27* colonizes the intestine to improve growth and barrier integrity in BALB/c mice and piglets. Diagram of the mice **(A)** and piglets **(D)** oral administration protocol. Quantification of 3 × pBD1/*ΔHLJ-27* in intestinal mucus of mice **(B)** and piglets **(E)** using real-time PCR. **C**. Intestinal morphology of ileum shown by H&E staining in mice. **F.** Intestinal morphology of ileum shown by H&E staining in piglets. S1A Fig created in BioRender. Yang, Y. Y. (2026) https://BioRender.com/3718cv1. S1D Fig created in BioRender. Yang, Y. Y. (2026) https://BioRender.com/66h7el8. Note: Different letters (a vs. b, a vs. c, a vs. d, b vs. c, b vs. d, c vs. d) denote statistically significant differences (*P* < 0.05).
(TIF)

S2 Fig The changes of intestinal microbial diversities in piglets with oral administration of 3 × pBD1/*ΔHLJ-27.* **A**. Rank abundance curves. **B**. PCoA of the gut microbiome composition on the species level among groups. **C**. ROC curve. **D**. Venn diagram of gut microbial composition. **E**. LEfSe analysis identifies microbial taxa, ranging from phylum to species level, that exhibit significant differences in abundance among the groups. **F**, **G** Predicted metabolic and KEGG pathway profiles of gut microbiota across different groups.
(TIF)

S3 Fig Detection of CD4^+^ T cell responses in PBMCs. (TIF)

S1 Table Primer sequence. (DOCX)

S2 Table Primer sequence. (DOCX)

S1 Raw Gel Uncropped original Western blot membranes. This file contains uncropped original Western blot membranes corresponding to the protein band data shown in Figs 1G and 7C of the main manuscript. All images were captured without adjustments to brightness, contrast or exposure.
(PDF)

## References

[1] Kirk MD, Pires SM, Black RE, Caipo M, Crump JA, Devleesschauwer B, et al. World Health Organization Estimates of the Global and Regional Disease Burden of 22 Foodborne Bacterial, Protozoal, and Viral Diseases, 2010: A Data Synthesis. PLoS Med. 2015;12(12):e1001921. doi: 10.1371/journal.pmed.1001921 PMC4668831 26633831

[2] Wang Z, Zhou H, Liu Y, Huang C, Chen J, Siddique A, et al. Nationwide trends and features of human salmonellosis outbreaks in China. Emerg Microbes Infect. 2024;13(1):2372364. doi: 10.1080/22221751.2024.2372364 38923510 PMC11259058

[3] Chen J, Huang L, An H, Wang Z, Kang X, Yin R, et al. One Health approach probes zoonotic non-typhoidal Salmonella infections in China: A systematic review and meta-analysis. J Glob Health. 2024;14:04256. doi: 10.7189/jogh.14.04256 39620281 PMC11610537

[4] Wang Y, Liu Y, Lyu N, Li Z, Ma S, Cao D, et al. The temporal dynamics of antimicrobial-resistant Salmonella enterica and predominant serovars in China. Natl Sci Rev. 2022;10(3):nwac269. doi: 10.1093/nsr/nwac269 37035020 PMC10076184

[5] Horne M, Woolley I, Lau JSY. The use of long-term antibiotics for suppression of bacterial infections. Clin Infect Dis. 2024;79(4):848–54. doi: 10.1093/cid/ciae302 38832929 PMC11478772

[6] Wang Y, Xu X, Jia S, Qu M, Pei Y, Qiu S, et al. A global atlas and drivers of antimicrobial resistance in Salmonella during 1900-2023. Nat Commun. 2025;16(1):4611. doi: 10.1038/s41467-025-59758-3 40382325 PMC12085583

[7] Jia C, Zhou H, Cao Q, Huang C, An H, Li Y, et al. Distinct genomic trajectory among invasive *Salmonella* Typhimurium ST313 infections. Nat Commun. 2026;17(1):3693. doi: 10.1038/s41467-026-70196-7 41803129 PMC13100060

[8] Zhou H, Jia C, Cao Q, Huang L, Teng L, Wang Z, et al. Invasive non-typhoidal Salmonella infections in China (1961-2024): a retrospective systematic analysis of multicentre case reports. J Glob Health. 2026;16:04008. doi: 10.7189/jogh.16.04008 41524234 PMC12796866

[9] Whitmore M, Tobin I, Burkardt A, Zhang G. Nutritional modulation of host defense peptide synthesis: a novel host-directed antimicrobial therapeutic strategy?. Adv Nutr. 2024;15(9):100277. doi: 10.1016/j.advnut.2024.100277 39053604 PMC11381887

[10] Andersson DI, Hughes D, Kubicek-Sutherland JZ. Mechanisms and consequences of bacterial resistance to antimicrobial peptides. Drug Resist Updat. 2016;26:43–57. doi: 10.1016/j.drup.2016.04.002 27180309

[11] Luo G, Zhang J, Wang H, Sun Y, Cheng B, Xu Z, et al. Human defensin-inspired discovery of peptidomimetic antibiotics. Proc Natl Acad Sci U S A. 2022;119(10):e2117283119. doi: 10.1073/pnas.2117283119 35238683 PMC8916014

[12] Chorawala MR, Chauhan S, Patel R, Shah G. Cell Wall Contents of Probiotics (Lactobacillus species) Protect Against Lipopolysaccharide (LPS)-Induced Murine Colitis by Limiting Immuno-inflammation and Oxidative Stress. Probiotics Antimicrob Proteins. 2021;13(4):1005–17. doi: 10.1007/s12602-020-09738-4 33544362

[13] Ganz T. Defensins: antimicrobial peptides of innate immunity. Nat Rev Immunol. 2003;3(9):710–20. doi: 10.1038/nri1180 12949495

[14] Selsted ME, Ouellette AJ. Mammalian defensins in the antimicrobial immune response. Nat Immunol. 2005;6(6):551–7. doi: 10.1038/ni1206 15908936

[15] Klotman ME, Chang TL. Defensins in innate antiviral immunity. Nat Rev Immunol. 2006;6(6):447–56. doi: 10.1038/nri1860 16724099

[16] Franzosa EA, Sirota-Madi A, Avila-Pacheco J, Fornelos N, Haiser HJ, Reinker S, et al. Gut microbiome structure and metabolic activity in inflammatory bowel disease. Nat Microbiol. 2019;4(2):293–305. doi: 10.1038/s41564-018-0306-4 30531976 PMC6342642

[17] Castellone V, Bancalari E, Rubert J, Gatti M, Neviani E, Bottari B. Eating fermented: health benefits of LAB-fermented foods. Foods. 2021;10(11). doi: 10.3390/foods10112639 PMC8620815 34828920

[18] De Filippis F, Pasolli E, Ercolini D. The food-gut axis: lactic acid bacteria and their link to food, the gut microbiome and human health. FEMS Microbiol Rev. 2020;44(4):454–89. doi: 10.1093/femsre/fuaa015 32556166 PMC7391071

[19] da Silva TF, Glória RA, Americo MF, Freitas ADS, de Jesus LCL, Barroso FAL. Unlocking the potential of probiotics: a comprehensive review on research, production, and regulation of probiotics. Probiotics Antimicrob Proteins. 2024;16(5):1687–723. doi: 10.1007/s12602-024-10247-x 38539008

[20] La Fata G, Weber P, Mohajeri MH. Probiotics and the gut immune system: indirect regulation. Probiotics Antimicrob Proteins. 2018;10(1):11–21. doi: 10.1007/s12602-017-9322-6 28861741 PMC5801397

[21] Yue S, Zhang Z, Bian F, Zhang Y, Chen G, Zhu Y, et al. 3L, three-lactobacilli on recovering of microbiome and immune-damage by cyclophosphamide chemotherapy—a pilot experiment in rats. Microbiology Research. 2023;14(3):831–69. doi: 10.3390/microbiolres14030059

[22] Kilic G, Yucel Sengun I. Multifunctional properties of vinegar-derived acetic acid bacteria and lactic acid bacteria: innovative potential probiotics. J Food Sci. 2026;91(1):e70775. doi: 10.1111/1750-3841.70775 41485215

[23] Shen J, Zhang J, Zhao Y, Lin Z, Ji L, Ma X. Tibetan pig-derived probiotic *Lactobacillus* amylovorus SLZX20-1 improved intestinal function via producing enzymes and regulating intestinal microflora. Front Nutr. 2022;9:846991. doi: 10.3389/fnut.2022.846991 35425795 PMC9002122

[24] Yue S, Ge H, Zou XT, Chen X, Wang C, Hu WC. A lactobacillus cocktail changes gut flora and reduces cholesterolemia and weight gain in hyperlipidemia mice. 2014.

[25] Yue S, He Q, Picimbon J-F. *Lactobacillus* for ribosome peptide editing cancer. Clin Transl Oncol. 2023;25(6):1522–44. doi: 10.1007/s12094-022-03066-5 36694080 PMC9873400

[26] Levit R, Cortes-Perez NG, de Moreno de Leblanc A, Loiseau J, Aucouturier A, Langella P, et al. Use of genetically modified lactic acid bacteria and bifidobacteria as live delivery vectors for human and animal health. Gut Microbes. 2022;14(1):2110821. doi: 10.1080/19490976.2022.2110821 35960855 PMC9377234

[27] Wells JM, Mercenier A. Mucosal delivery of therapeutic and prophylactic molecules using lactic acid bacteria. Nat Rev Microbiol. 2008;6(5):349–62. doi: 10.1038/nrmicro1840 18345021 PMC7096801

[28] Yue S, Li Z, Hu F, Picimbon J-F. Curing piglets from diarrhea and preparation of a healthy microbiome with Bacillus treatment for industrial animal breeding. Sci Rep. 2020;10(1):19476. doi: 10.1038/s41598-020-75207-1 33173074 PMC7656456

[29] Lee N, Cozzitorto J, Wainwright N, Testa D. Cloning with tandem gene systems for high level gene expression. Nucleic Acids Res. 1984;12(17):6797–812. doi: 10.1093/nar/12.17.6797 6091039 PMC320117

[30] Lee JH, Kim MS, Cho JH, Kim SC. Enhanced expression of tandem multimers of the antimicrobial peptide buforin II in Escherichia coli by the DEAD-box protein and trxB mutant. Appl Microbiol Biotechnol. 2002;58(6):790–6. doi: 10.1007/s00253-002-0962-3 12021800

[31] Mansur M, Cabello C, Hernández L, País J, Varas L, Valdés J, et al. Multiple gene copy number enhances insulin precursor secretion in the yeast Pichia pastoris. Biotechnol Lett. 2005;27(5):339–45. doi: 10.1007/s10529-005-1007-7 15834796

[32] Acurcio LB, Wuyts S, de Cicco Sandes SH, Sant’anna FM, Pedroso SHSP, Bastos RW, et al. Milk fermented by *Lactobacillus* paracasei NCC 2461 (ST11) modulates the immune response and microbiota to exert its protective effects against salmonella typhimurium infection in mice. Probiotics Antimicrob Proteins. 2020;12(4):1398–408. doi: 10.1007/s12602-020-09634-x 31970649

[33] Muyyarikkandy MS, Amalaradjou MA. Lactobacillus bulgaricus, *Lactobacillus rhamnosus* and *Lactobacillus paracasei* attenuate Salmonella enteritidis, *Salmonella* heidelberg and *Salmonella* typhimurium colonization and virulence gene expression in vitro. Int J Mol Sci. 2017;18(11):12381. doi: 10.3390/ijms18112381 PMC5713350 29120368

[34] Floor E, Su J, Chatterjee M, Kuipers ES, IJssennagger N, Heidari F, et al. Development of a Caco-2-based intestinal mucosal model to study intestinal barrier properties and bacteria-mucus interactions. Gut Microbes. 2025;17(1):2434685. doi: 10.1080/19490976.2024.2434685 39714032 PMC11702969

[35] Liu Z, Zhou X, Wang W, Gu L, Hu C, Sun H, et al. *Lactobacillus paracasei* 24 attenuates lipid accumulation in high-fat diet-induced obese mice by regulating the gut microbiota. J Agric Food Chem. 2022;70(15):4631–43. doi: 10.1021/acs.jafc.1c07884 35377154

[36] Zhu J, Gao M, Zhang R, Sun Z, Wang C, Yang F, et al. Effects of soybean meal fermented by L. plantarum, B. subtilis and S. cerevisieae on growth, immune function and intestinal morphology in weaned piglets. Microb Cell Fact. 2017;16(1):191. doi: 10.1186/s12934-017-0809-3 29121938 PMC5679485

[37] Santamaria JC, Borelli A, Irla M. Regulatory T cell heterogeneity in the thymus: impact on their functional activities. Front Immunol. 2021;12:643153. doi: 10.3389/fimmu.2021.643153 33643324 PMC7904894

[38] Zhang J, Tang C, Liu Y, Sun J, Li X, Long K, et al. Single cell transcriptome profiling of immune tissues from germ-free and specific pathogen-free piglet. Sci Data. 2025;12(1):652. doi: 10.1038/s41597-025-04957-2 40251240 PMC12008294

[39] Guo J, Zhao Y, Guo W, Sun Y, Zhang W, Zhao Q, et al. Effects of *Lactobacillus paracei* JY062 postbiotic on intestinal barrier, immunity, and gut microbiota. Nutrients. 2025;17(7):1272. doi: 10.3390/nu17071272 40219029 PMC11990213

[40] Han F, Zhang H, Xia X, Xiong H, Song D, Zong X, et al. Porcine β-defensin 2 attenuates inflammation and mucosal lesions in dextran sodium sulfate-induced colitis. J Immunol. 2015;194(4):1882–93. doi: 10.4049/jimmunol.1402300 25601921

[41] Tang Q, Xu E, Wang Z, Xiao M, Cao S, Hu S, et al. Dietary hermetia illucens larvae meal improves growth performance and intestinal barrier function of weaned pigs under the environment of enterotoxigenic Escherichia coli K88. Front Nutr. 2022;8:812011. doi: 10.3389/fnut.2021.812011 35118109 PMC8805673

[42] Xu J, Jia Z, Xiao S, Long C, Wang L. Effects of Enterotoxigenic Escherichia coli Challenge on Jejunal Morphology and Microbial Community Profiles in Weaned Crossbred Piglets. Microorganisms. 2023;11(11):2646. doi: 10.3390/microorganisms11112646 38004658 PMC10672776

[43] Peng X, Wang R, Hu L, Zhou Q, Liu Y, Yang M, et al. Enterococcus faecium NCIMB 10415 administration improves the intestinal health and immunity in neonatal piglets infected by enterotoxigenic Escherichia coli K88. J Anim Sci Biotechnol. 2019;10:72. doi: 10.1186/s40104-019-0376-z 31452881 PMC6702752

[44] Du M, Liu X, Ji X, Wang Y, Liu X, Zhao C, et al. Berberine alleviates enterotoxigenic Escherichia coli-induced intestinal mucosal barrier function damage in a piglet model by modulation of the intestinal microbiome. Front Nutr. 2025;11:1494348. doi: 10.3389/fnut.2024.1494348 39877539 PMC11772193

[45] Yu HT, Ding XL, Li N, Zhang XY, Zeng XF, Wang S, et al. Dietary supplemented antimicrobial peptide microcin J25 improves the growth performance, apparent total tract digestibility, fecal microbiota, and intestinal barrier function of weaned pigs. J Anim Sci. 2017;95(11):5064–76. doi: 10.2527/jas2017.1494 29293710 PMC6292272

[46] Barbara G, Barbaro MR, Fuschi D, Palombo M, Falangone F, Cremon C, et al. Inflammatory and microbiota-related regulation of the intestinal epithelial barrier. Front Nutr. 2021;8:718356. doi: 10.3389/fnut.2021.718356 34589512 PMC8475765

[47] Keestra-Gounder AM, Tsolis RM, Bäumler AJ. Now you see me, now you don’t: the interaction of Salmonella with innate immune receptors. Nat Rev Microbiol. 2015;13(4):206–16. doi: 10.1038/nrmicro3428 25749454

[48] Jones BD, Ghori N, Falkow S. Salmonella typhimurium initiates murine infection by penetrating and destroying the specialized epithelial M cells of the Peyer’s patches. J Exp Med. 1994;180(1):15–23. doi: 10.1084/jem.180.1.15 8006579 PMC2191576

[49] Haraga A, Ohlson MB, Miller SI. *Salmonellae* interplay with host cells. Nat Rev Microbiol. 2008;6(1):53–66. doi: 10.1038/nrmicro1788 18026123

[50] LaRock DL, Chaudhary A, Miller SI. *Salmonellae* interactions with host processes. Nat Rev Microbiol. 2015;13(4):191–205. doi: 10.1038/nrmicro3420 25749450 PMC5074537

[51] Liang H, Dai Z, Liu N, Ji Y, Chen J, Zhang Y, et al. Dietary L-tryptophan modulates the structural and functional composition of the intestinal microbiome in weaned piglets. Front Microbiol. 2018;9:1736. doi: 10.3389/fmicb.2018.01736 30131777 PMC6090026

[52] Li Q, Zheng T, Ding H, Chen J, Li B, Zhang Q, et al. Exploring the benefits of probiotics in gut inflammation and diarrhea-from an antioxidant perspective. Antioxidants (Basel). 2023;12(7):1342. doi: 10.3390/antiox12071342 37507882 PMC10376667

[53] Xu C, Guo Y, Qiao L, Ma L, Cheng Y, Roman A. Biogenic synthesis of novel functionalized selenium nanoparticles by *Lactobacillus casei* ATCC 393 and its protective effects on intestinal barrier dysfunction caused by enterotoxigenic Escherichia coli K88. Front Microbiol. 2018;9:1129. doi: 10.3389/fmicb.2018.01129 29967593 PMC6015882

[54] Sun T, Nguyen A, Gommerman JL. Dendritic cell subsets in intestinal immunity and inflammation. J Immunol. 2020;204(5):1075–83. doi: 10.4049/jimmunol.1900710 32071090

[55] Scott CL, Aumeunier AM, Mowat AM. Intestinal CD103+ dendritic cells: master regulators of tolerance?. Trends Immunol. 2011;32(9):412–9. doi: 10.1016/j.it.2011.06.003 21816673

[56] Yuan X, Wang J, Cheng M, Zhang X. Mouse β-defensin-14 for inducing the maturation of dendritic cells. Int Immunopharmacol. 2018;55:133–41. doi: 10.1016/j.intimp.2017.12.017 29253819

[57] Biragyn A, Ruffini PA, Leifer CA, Klyushnenkova E, Shakhov A, Chertov O, et al. Toll-like receptor 4-dependent activation of dendritic cells by beta-defensin 2. Science. 2002;298(5595):1025–9. doi: 10.1126/science.1075565 12411706

[58] Niyonsaba F, Ushio H, Nakano N, Ng W, Sayama K, Hashimoto K, et al. Antimicrobial peptides human beta-defensins stimulate epidermal keratinocyte migration, proliferation and production of proinflammatory cytokines and chemokines. J Invest Dermatol. 2007;127(3):594–604. doi: 10.1038/sj.jid.5700599 17068477

[59] Tang J, Zeng C, Cox TM, Li C, Son YM, Cheon IS, et al. Respiratory mucosal immunity against SARS-CoV-2 after mRNA vaccination. Sci Immunol. 2022;7(76):eadd4853. doi: 10.1126/sciimmunol.add4853 35857583 PMC9348751

[60] Rose-John S, Jenkins BJ, Garbers C, Moll JM, Scheller J. Targeting IL-6 trans-signalling: past, present and future prospects. Nat Rev Immunol. 2023;23(10):666–81. doi: 10.1038/s41577-023-00856-y 37069261 PMC10108826

[61] York AG, Skadow MH, Oh J, Qu R, Zhou QD, Hsieh W-Y, et al. IL-10 constrains sphingolipid metabolism to limit inflammation. Nature. 2024;627(8004):628–35. doi: 10.1038/s41586-024-07098-5 38383790 PMC10954550

[62] Kim JH, Kim DH, Jo S, Cho MJ, Cho YR, Lee YJ, et al. Immunomodulatory functional foods and their molecular mechanisms. Exp Mol Med. 2022;54(1):1–11. doi: 10.1038/s12276-022-00724-0 35079119 PMC8787967

[63] Hu J, Chen L, Zheng W, Shi M, Liu L, Xie C, et al. *Lactobacillus* frumenti facilitates intestinal epithelial barrier function maintenance in early-weaned piglets. Front Microbiol. 2018;9:897. doi: 10.3389/fmicb.2018.00897 29867808 PMC5958209

[64] Moniruzzaman M, Rahman MA, Wang R, Wong KY, Chen AC-H, Mueller A, et al. Interleukin-22 suppresses major histocompatibility complex II in mucosal epithelial cells. J Exp Med. 2023;220(11):e20230106. doi: 10.1084/jem.20230106 37695525 PMC10494524

[65] Fekete E, Allain T, Sosnowski O, Anderson S, Lewis IA, Buret AG. Giardia spp.-induced microbiota dysbiosis disrupts intestinal mucin glycosylation. Gut Microbes. 2024;16(1):2412676. doi: 10.1080/19490976.2024.2412676 39412866 PMC11485787

