# Supplementary material for: Engineered Lactobacillus paracasei 3×pBD1/ΔHLJ-27 as a novel antibiotic alternative for livestock: Dual efficacy in growth promotion and protection against Salmonella infection via integrated host-microbiota-immune regulation
Source: PLoS Pathog. 2026 Aug 14;22(8):e1014367. doi: 10.1371/journal.ppat.1014367 (PMC13475985; doi:10.1371/journal.ppat.1014367)
Supplement: Supplementary file 1 [file ppat.1014367.s006.pdf]

Figure1G

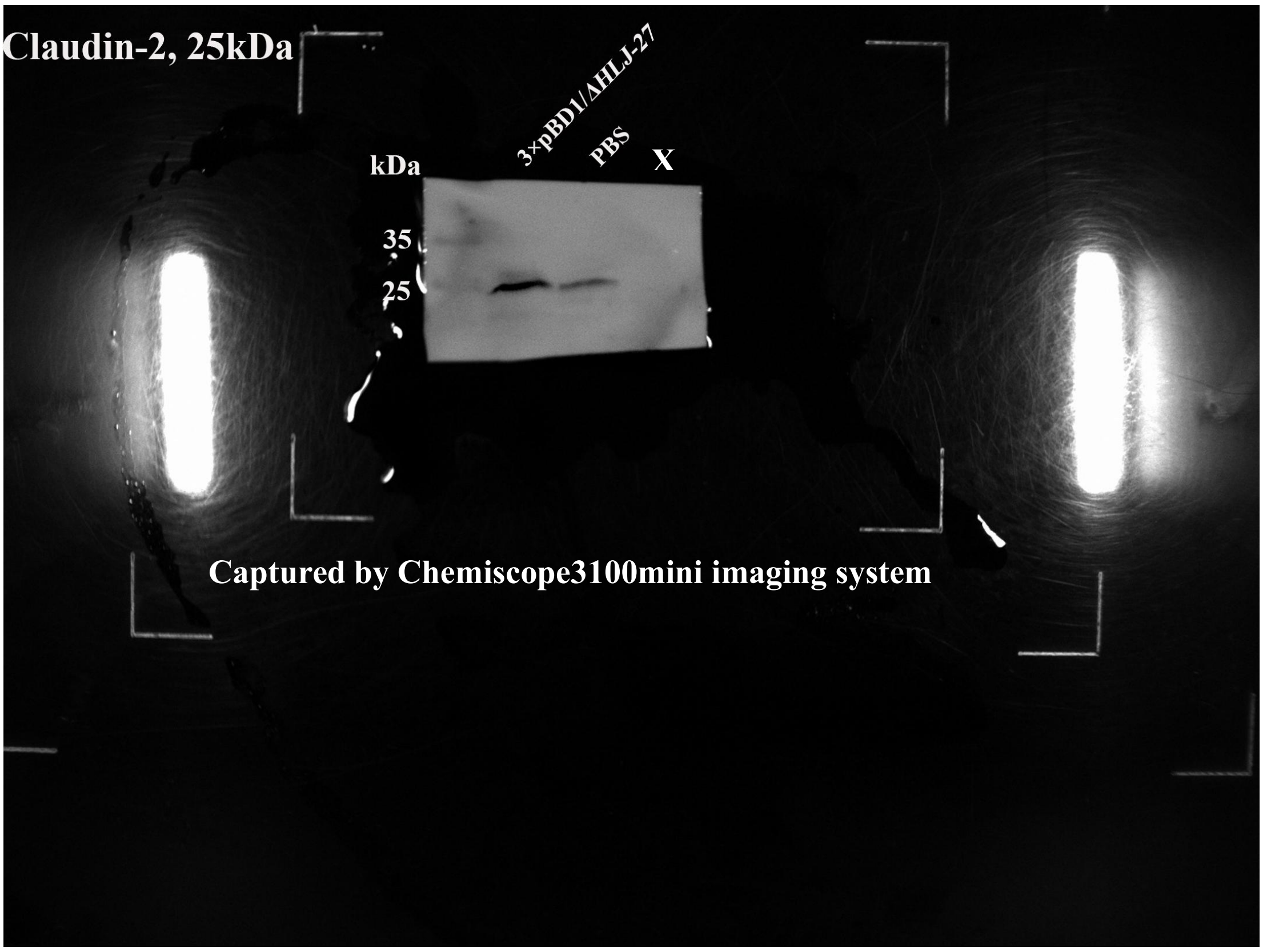
Claudin-2, 25kDa
kDa
3×pBD1/ΔHLJ-27
PBS
X
35
25
Captured by Chemiscope3100mini imaging system

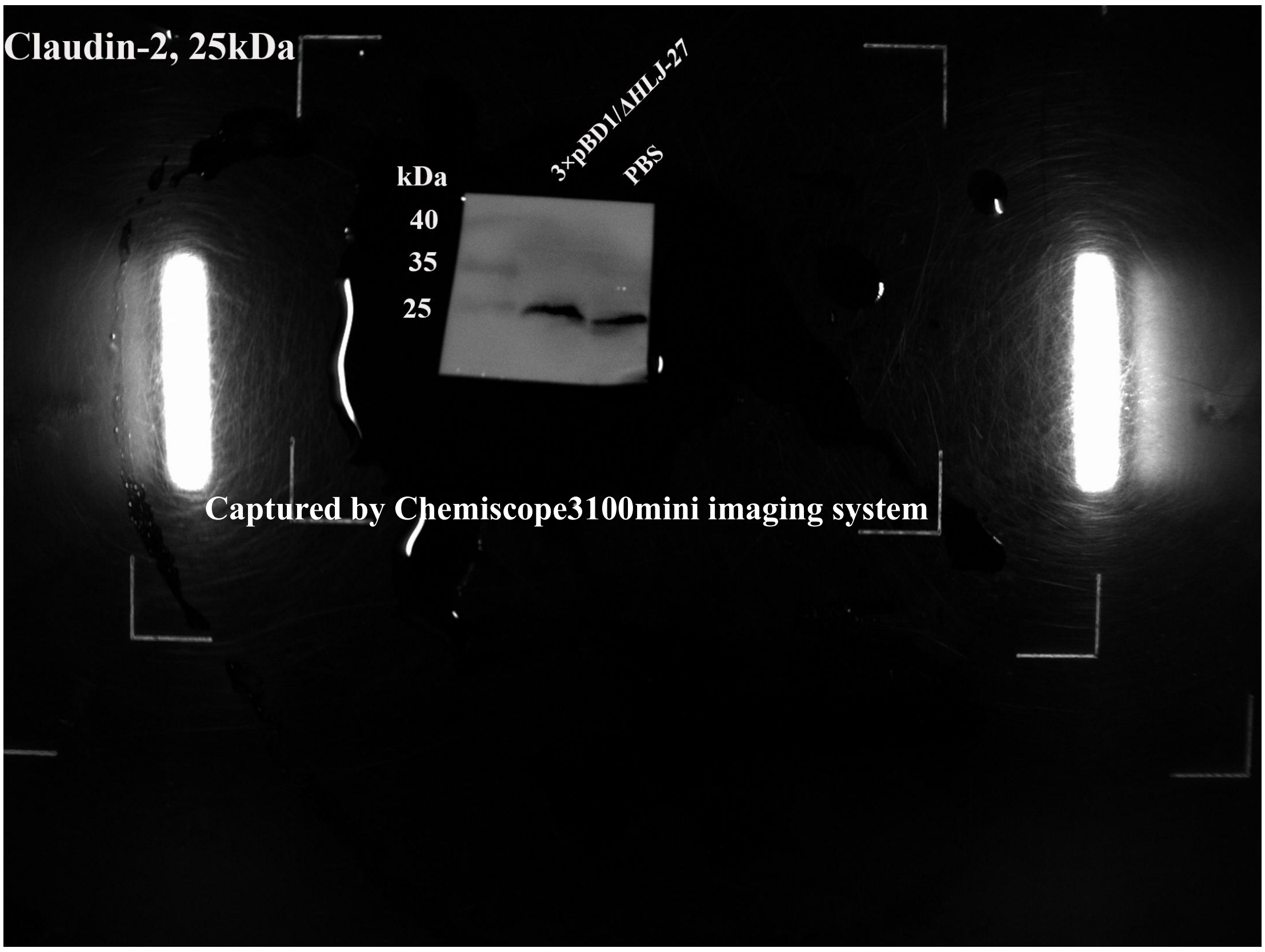
Claudin-2, 25kDa
kDa
3×pBD1/ΔHLJ-27
PBS
40
35
25
Captured by Chemiscope3100mini imaging system

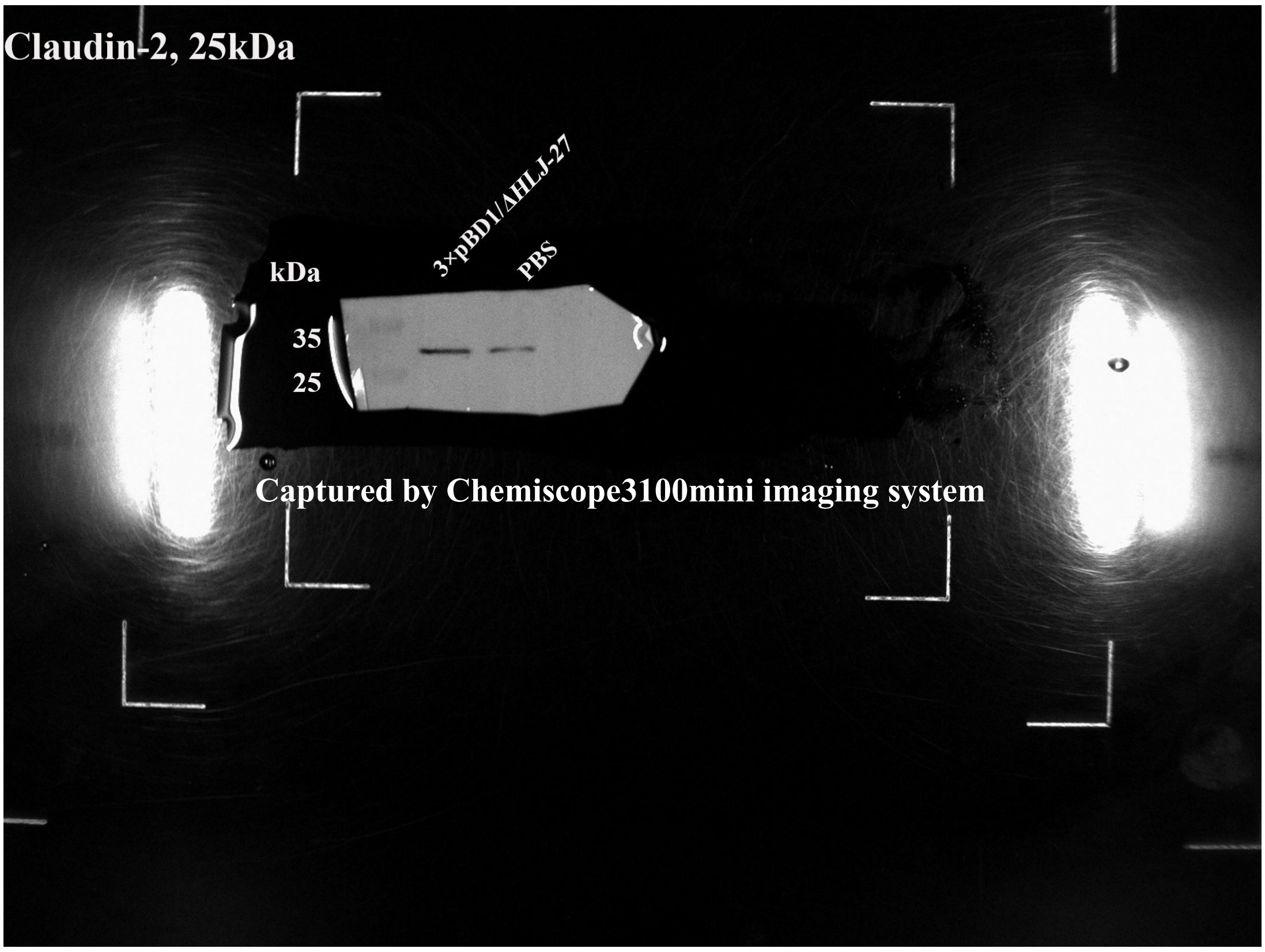
Claudin-2, 25kDa
kDa
3×pBD1/ΔHLJ-27
PBS
35
25
Captured by Chemiscope3100mini imaging system

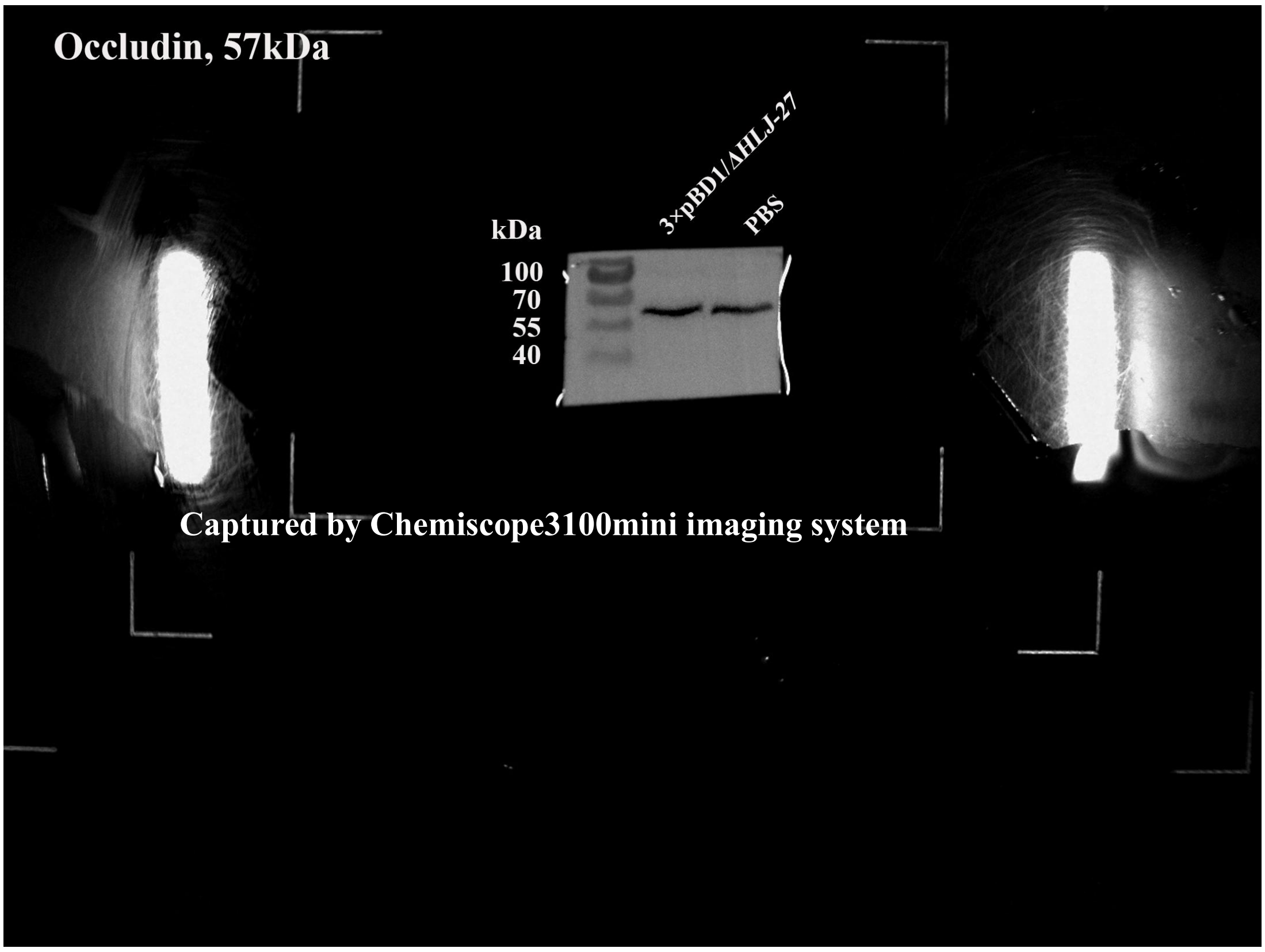
Occludin, 57kDa
3×pBD1/ΔHLJ-27
PBS
kDa
100
70
55
40
Captured by Chemiscope3100mini imaging system

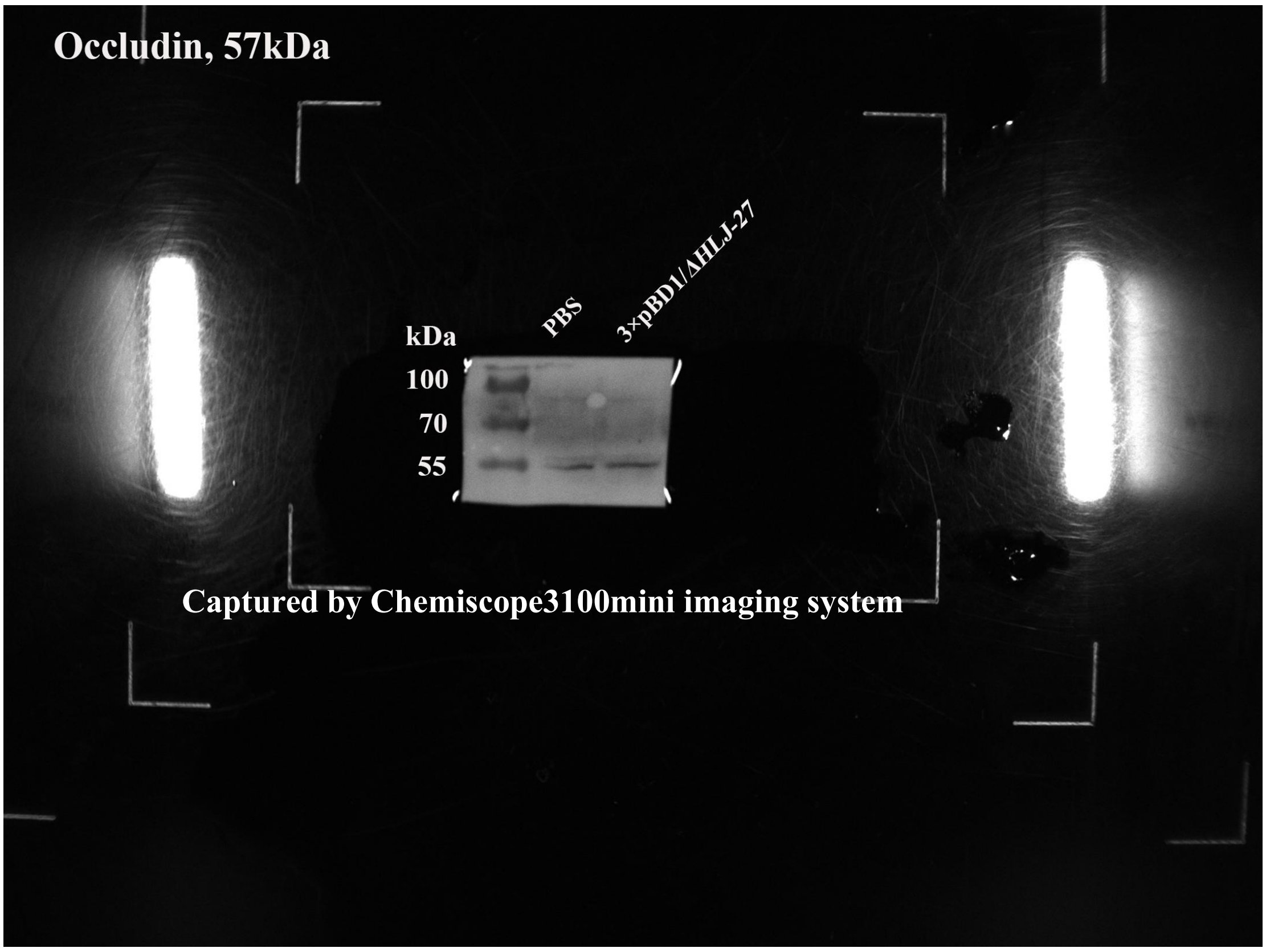
Occludin, 57kDa
PBS
3×pBD1/ΔHLJ-27
kDa
100
70
55
Captured by Chemiscope3100mini imaging system

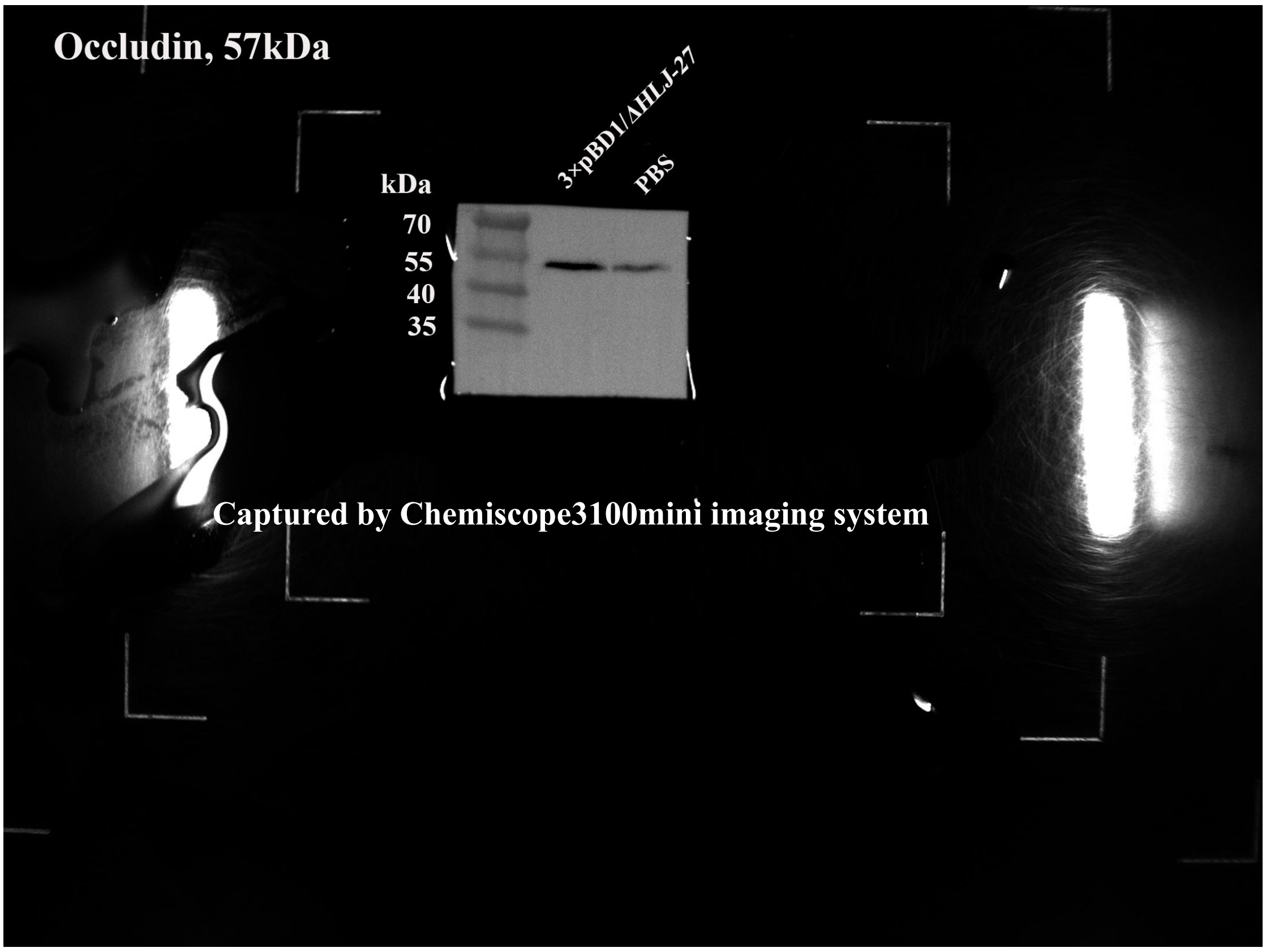
Occludin, 57kDa
3×pBD1/ΔHLJ-27
PBS
kDa
70
55
40
35
Captured by Chemiscope3100mini imaging system

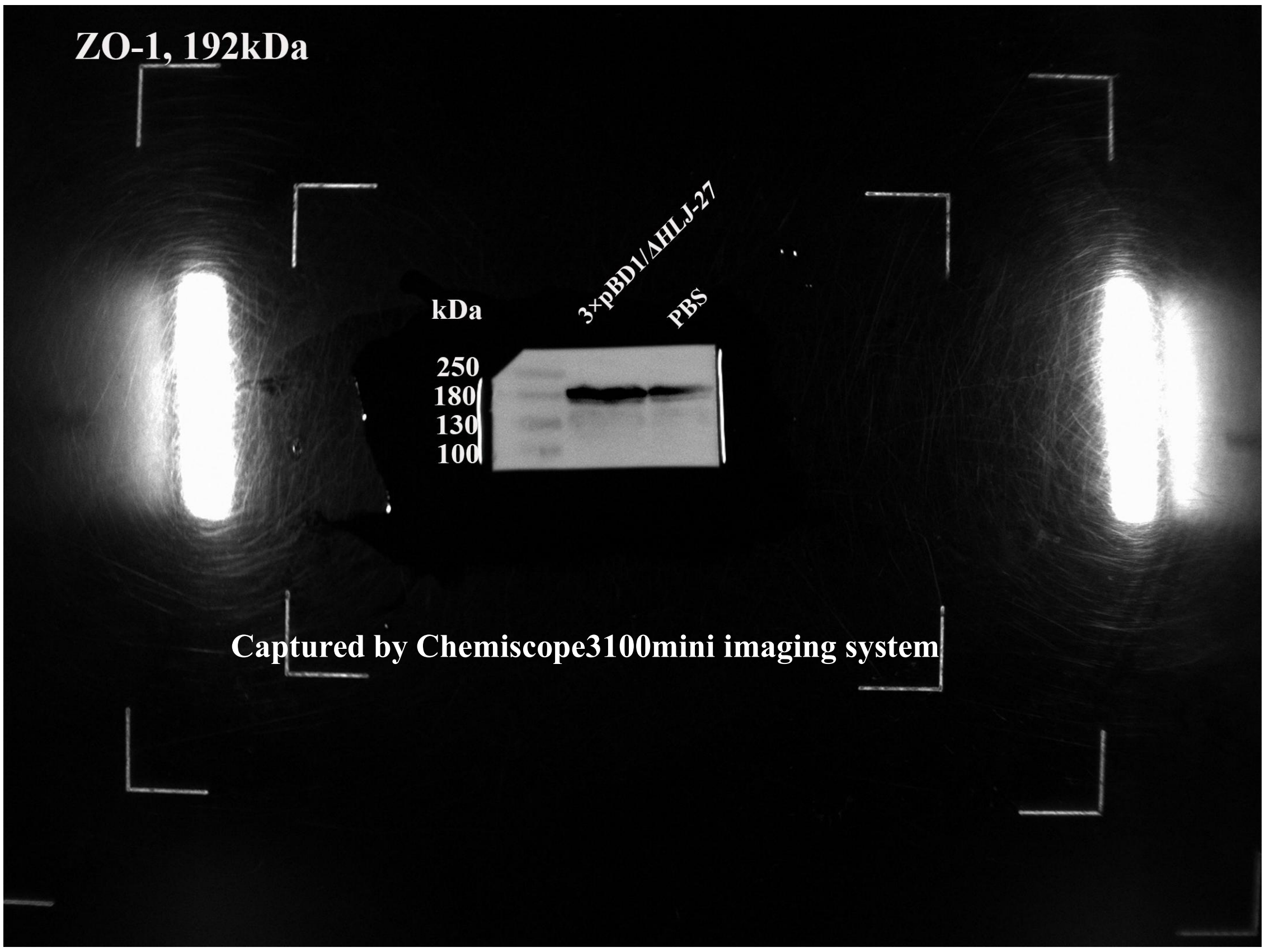
ZO-1, 192kDa
3×pBD1/ΔHLJ-27
PBS
kDa
250
180
130
100
Captured by Chemiscope3100mini imaging system

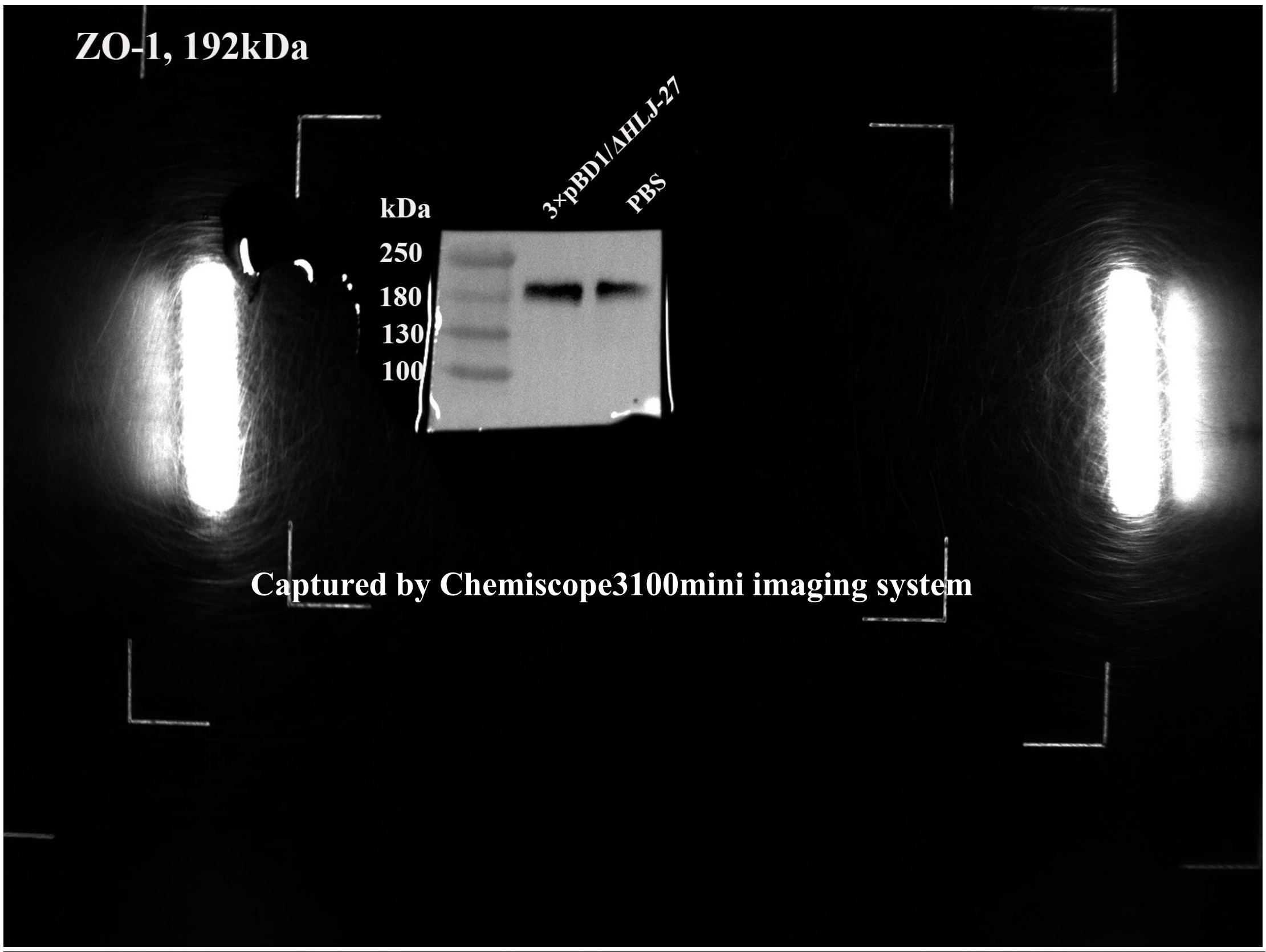
ZO-1, 192kDa
kDa
250
180
130
100
3×pBD1/ΔHLJ-27
PBS
Captured by Chemiscope3100mini imaging system

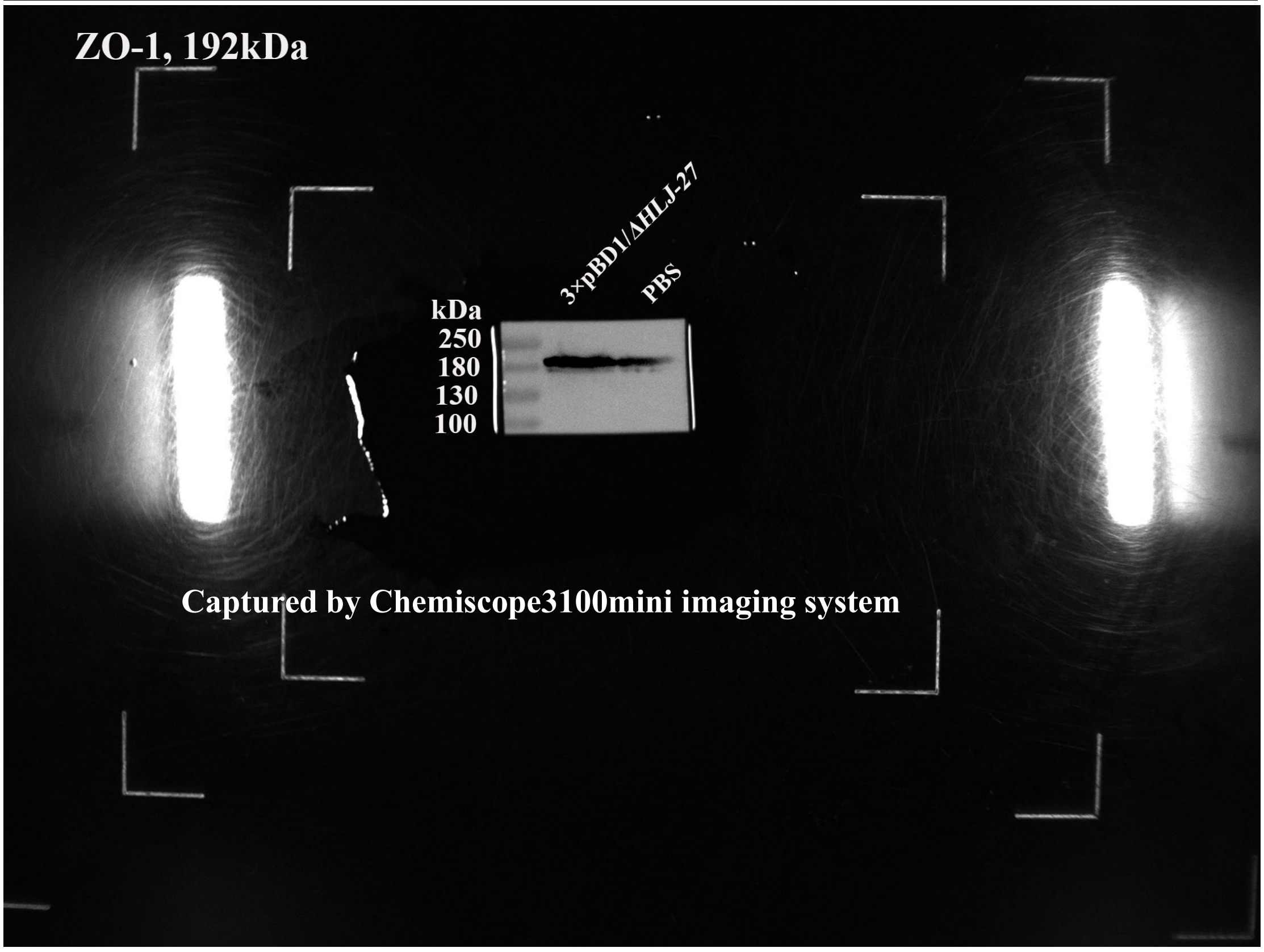
ZO-1, 192kDa
kDa
250
180
130
100
3×pBD1/ΔHLJ-27
PBS
Captured by Chemiscope3100mini imaging system

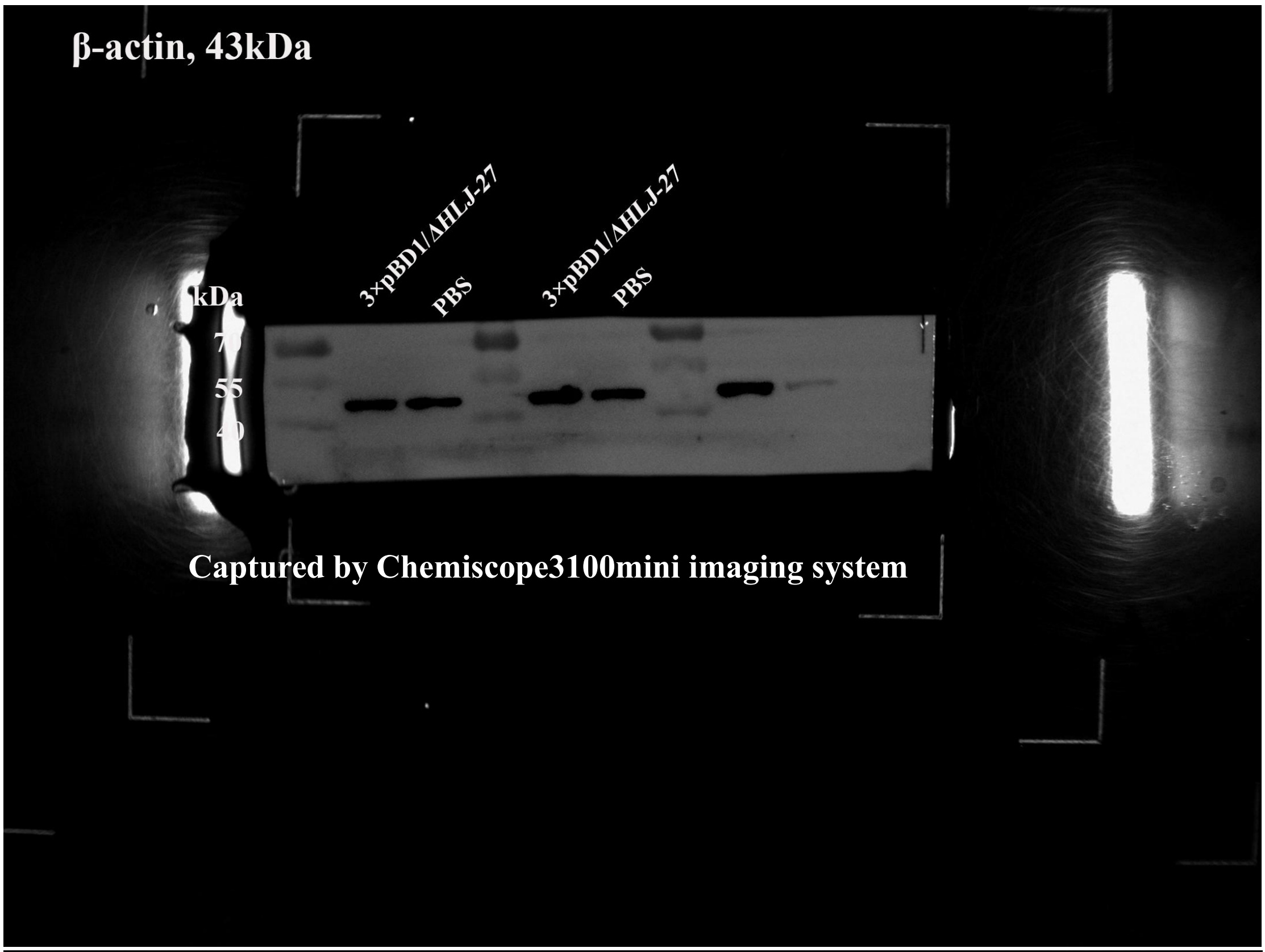
β-actin, 43kDa
kDa
70
55
40
3×pBD1/ΔHLJ-27
PBS
3×pBD1/ΔHLJ-27
PBS
Captured by Chemiscope3100mini imaging system

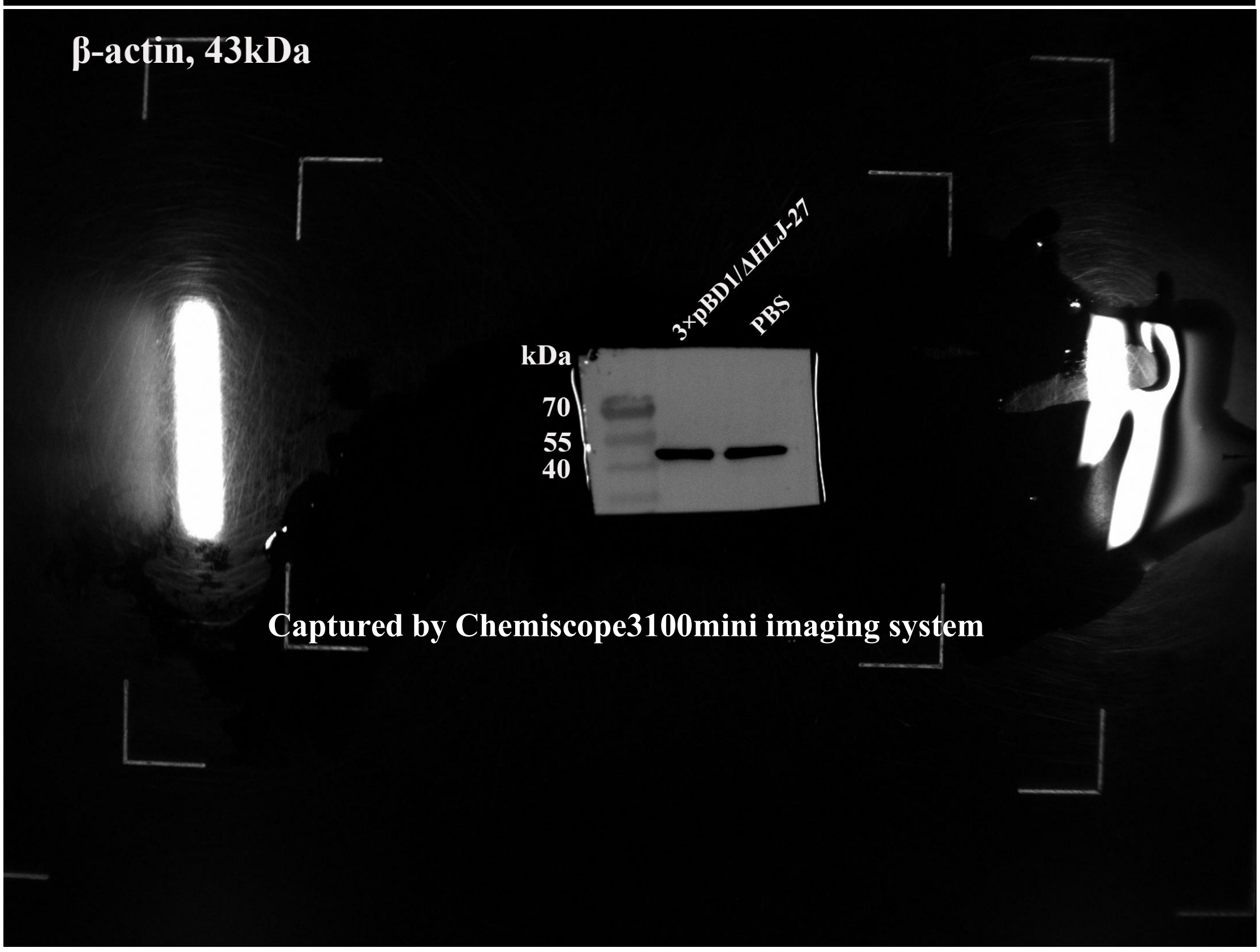
β-actin, 43kDa
kDa
70
55
40
3×pBD1/ΔHLJ-27
PBS
Captured by Chemiscope3100mini imaging system

Figure7C

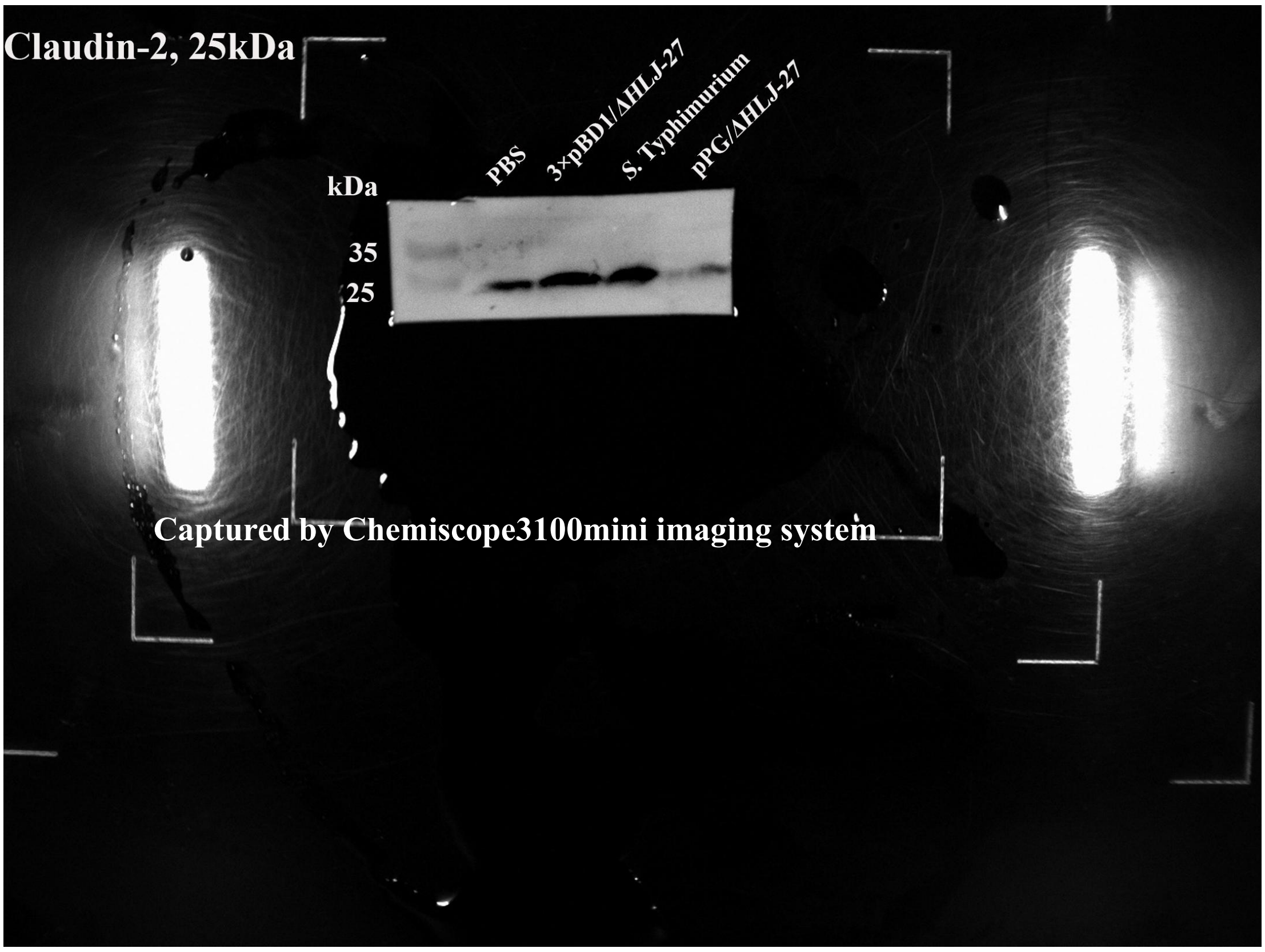

Claudin-2, 25kDa
PBS
3×pBD1/ΔHLJ-27
S. Typhimurium
pPG/ΔHLJ-27
kDa
35
25
Captured by Chemiscope3100mini imaging system

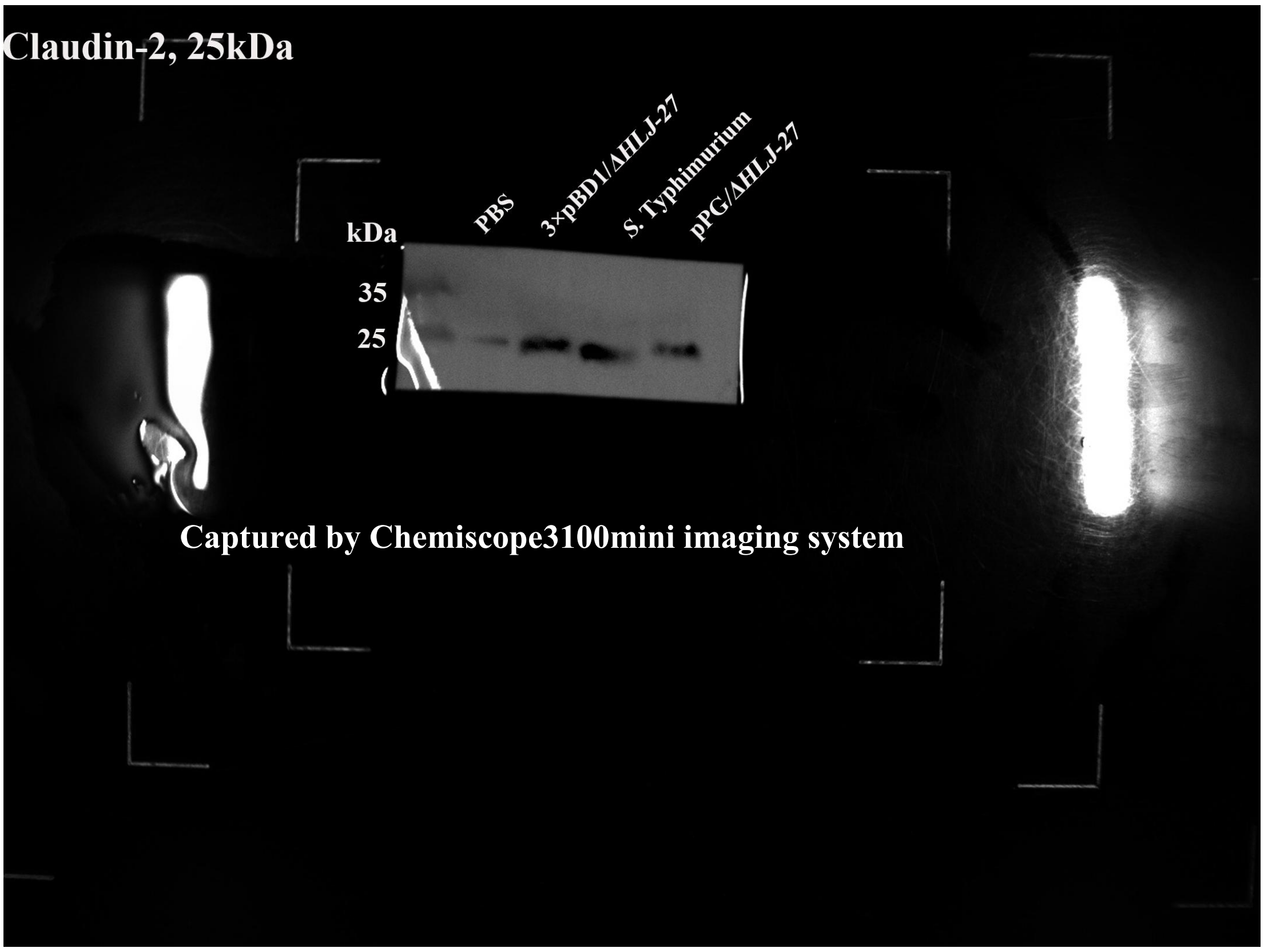

Claudin-2, 25kDa
PBS
3×pBD1/ΔHLJ-27
S. Typhimurium
pPG/ΔHLJ-27
kDa
35
25
Captured by Chemiscope3100mini imaging system

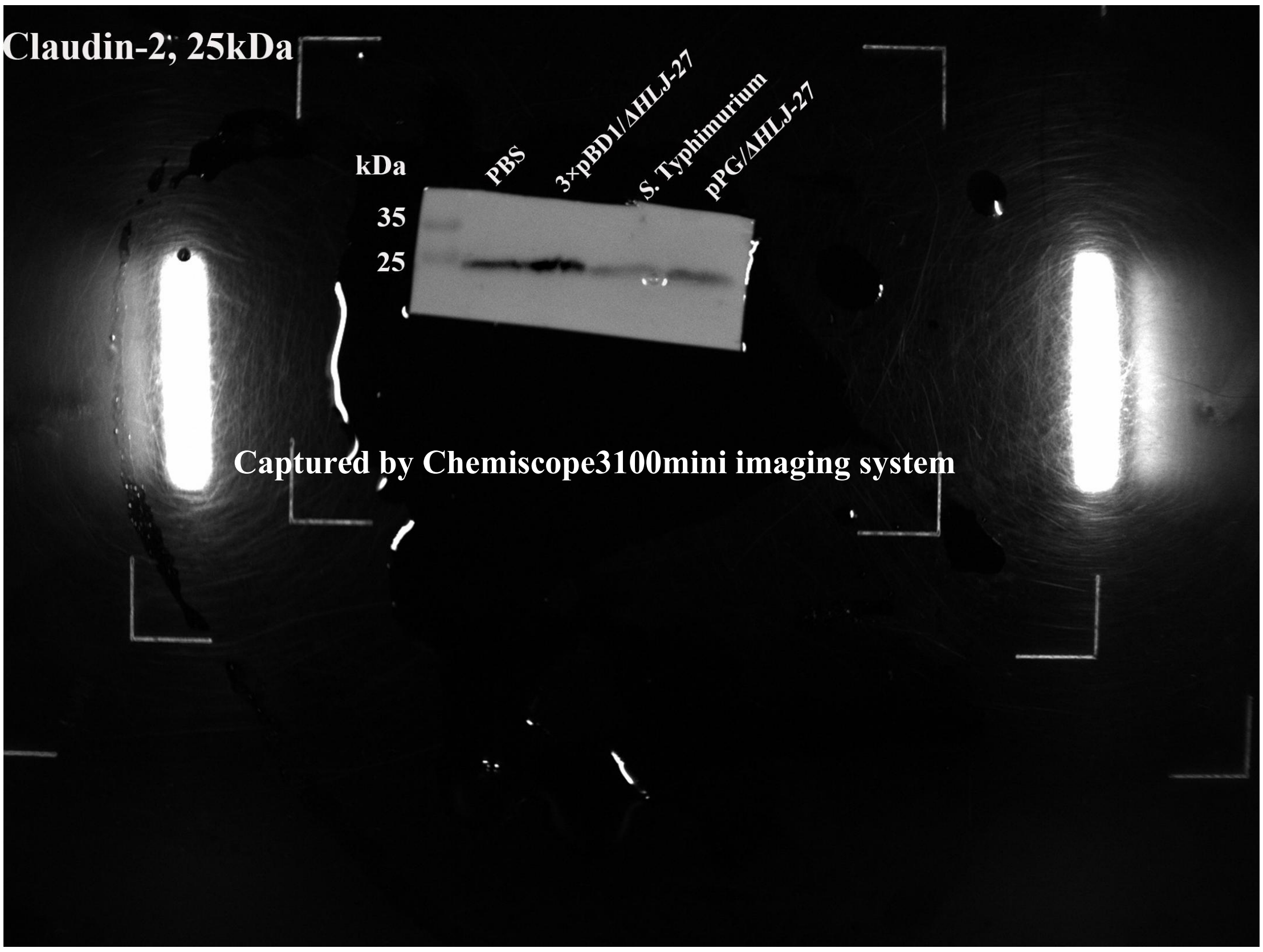
Claudin-2, 25kDa
kDa
35
25
PBS
3×pBD1/ΔHLJ-27
S. Typhimurium
pPG/ΔHLJ-27
Captured by Chemiscope3100mini imaging system

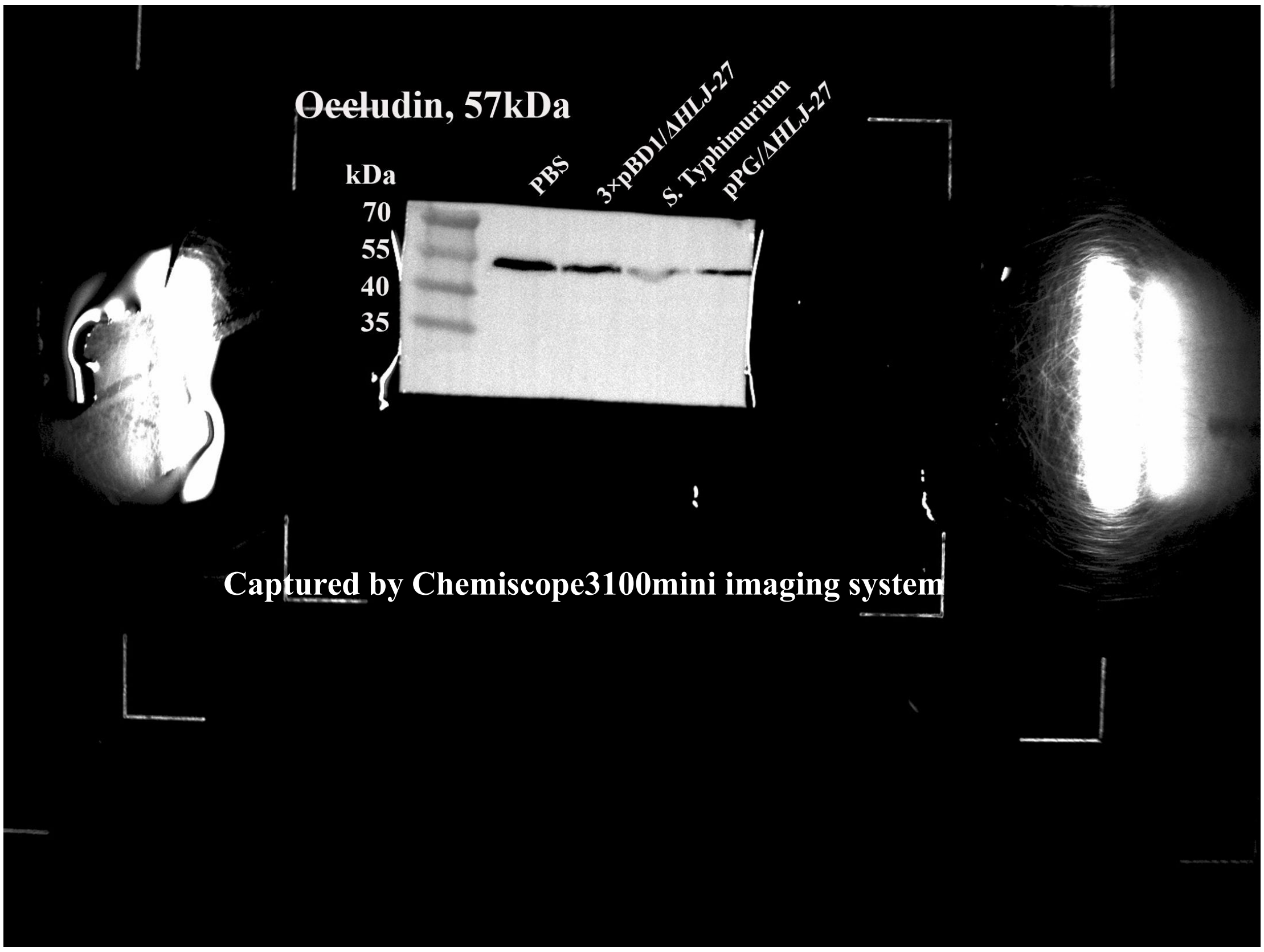
Occludin, 57kDa
kDa
70
55
40
35
PBS
3×pBD1/ΔHLJ-27
S. Typhimurium
pPG/ΔHLJ-27
Captured by Chemiscope3100mini imaging system

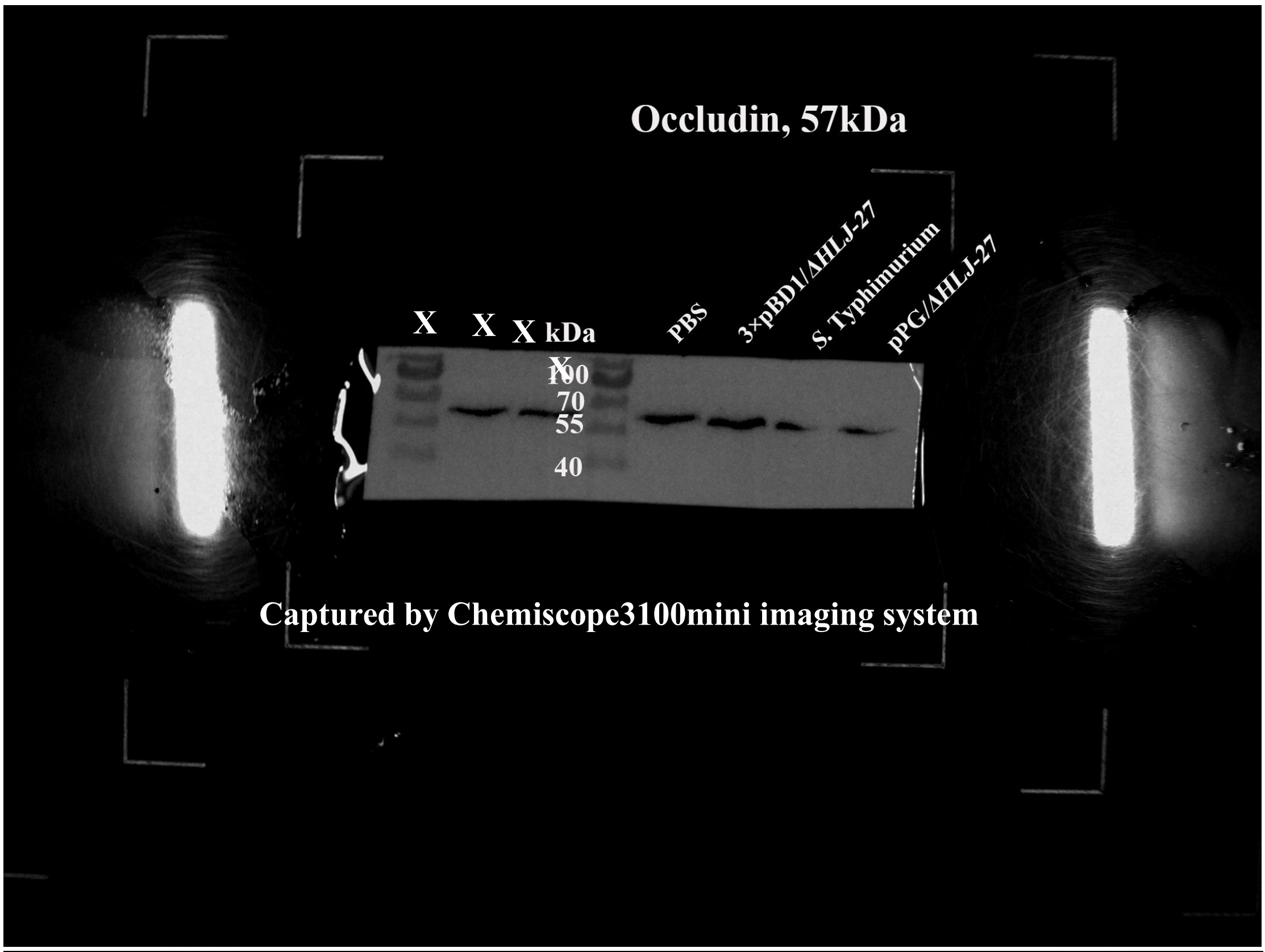
Occludin, 57kDa
X
X
X
X
kDa
100
70
55
40
PBS
3×pBD1/ΔHLJ-27
S. Typhimurium
pPG/ΔHLJ-27
Captured by Chemiscope3100mini imaging system

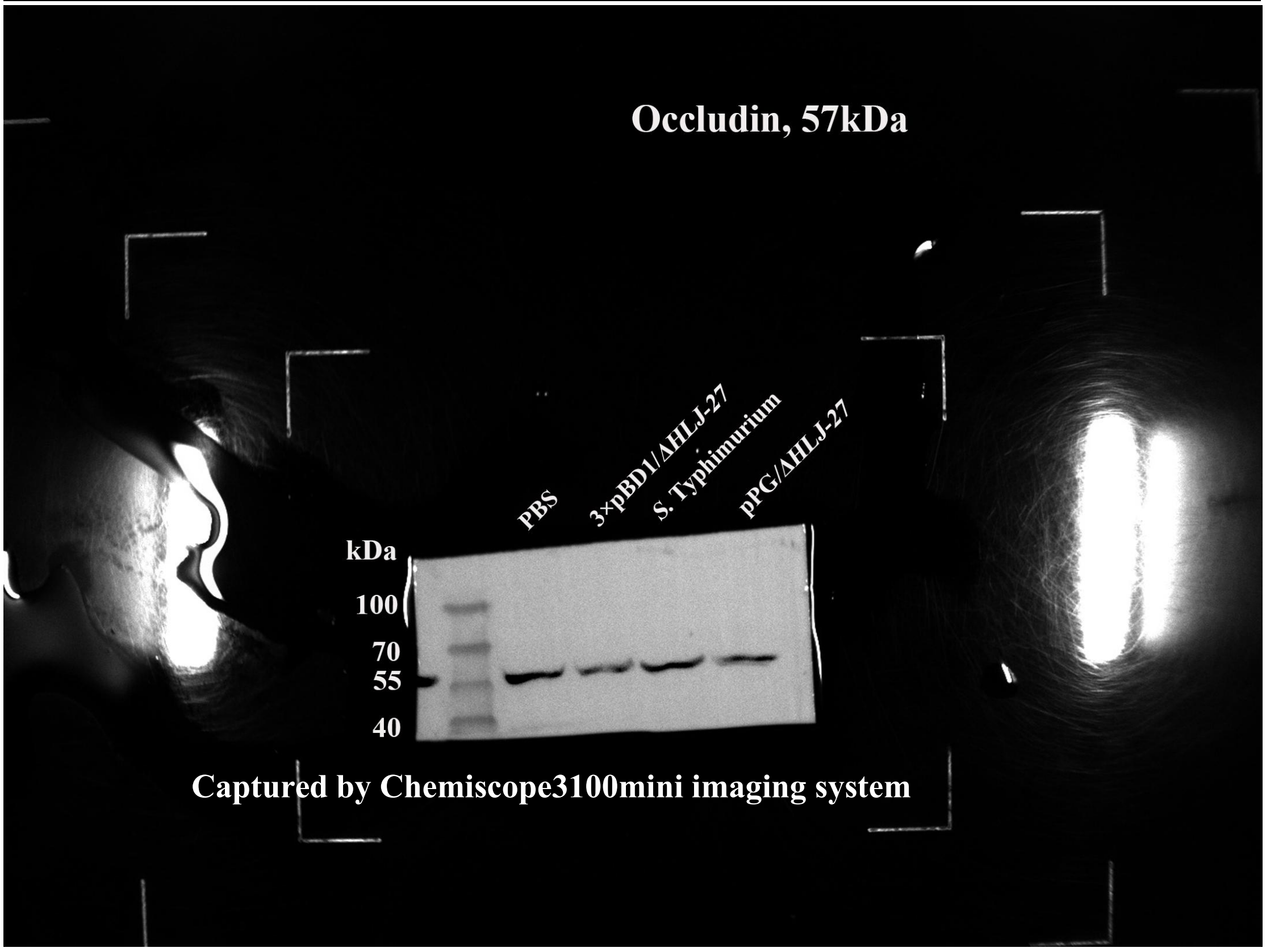
Occludin, 57kDa
PBS
3×pBD1/ΔHLJ-27
S. Typhimurium
pPG/ΔHLJ-27
kDa
100
70
55
40
Captured by Chemiscope3100mini imaging system

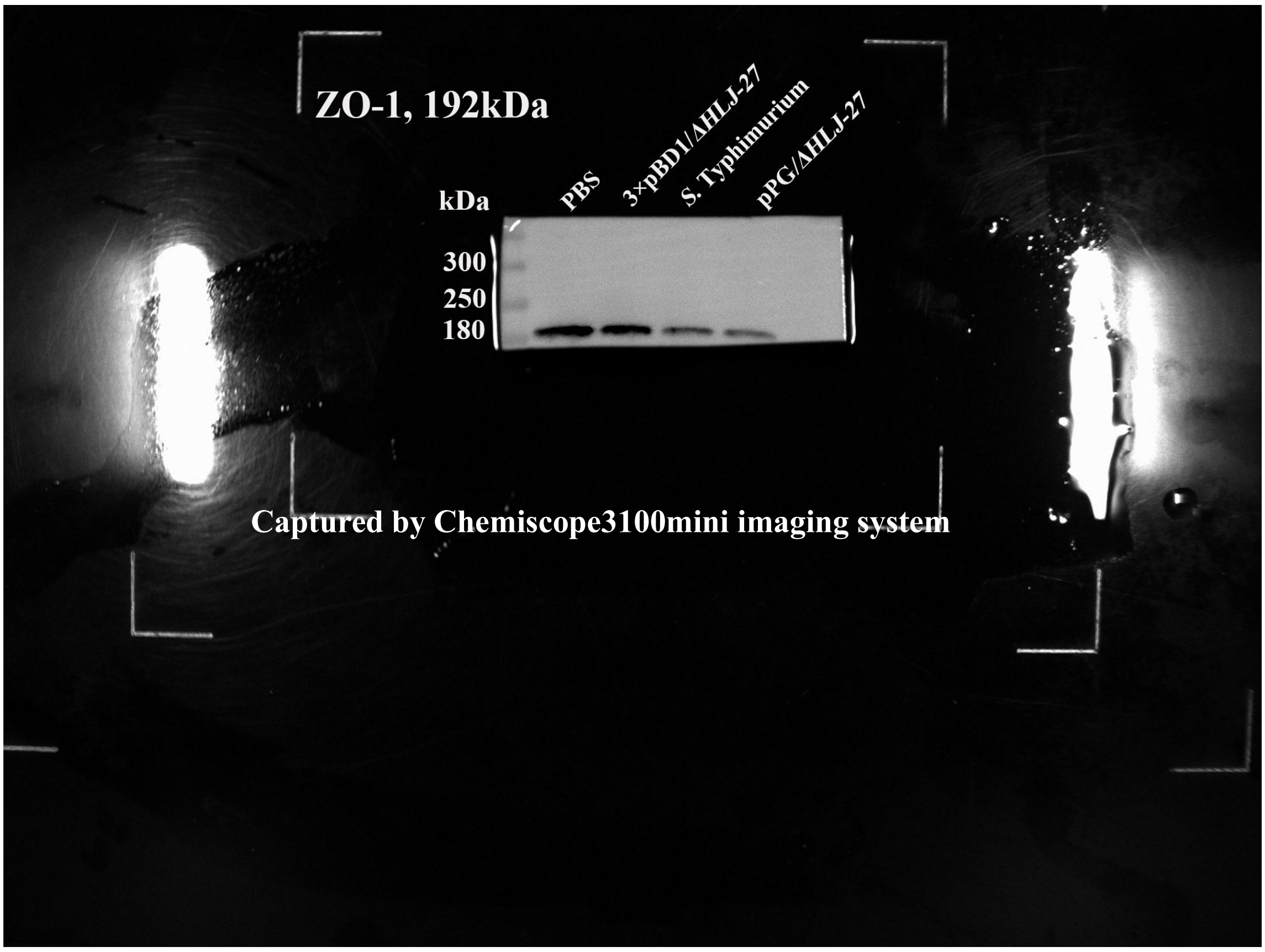
ZO-1, 192kDa
kDa
PBS
3×pBD1/ΔHLJ-27
S. Typhimurium
pPG/ΔHLJ-27
300
250
180
Captured by Chemiscope3100mini imaging system

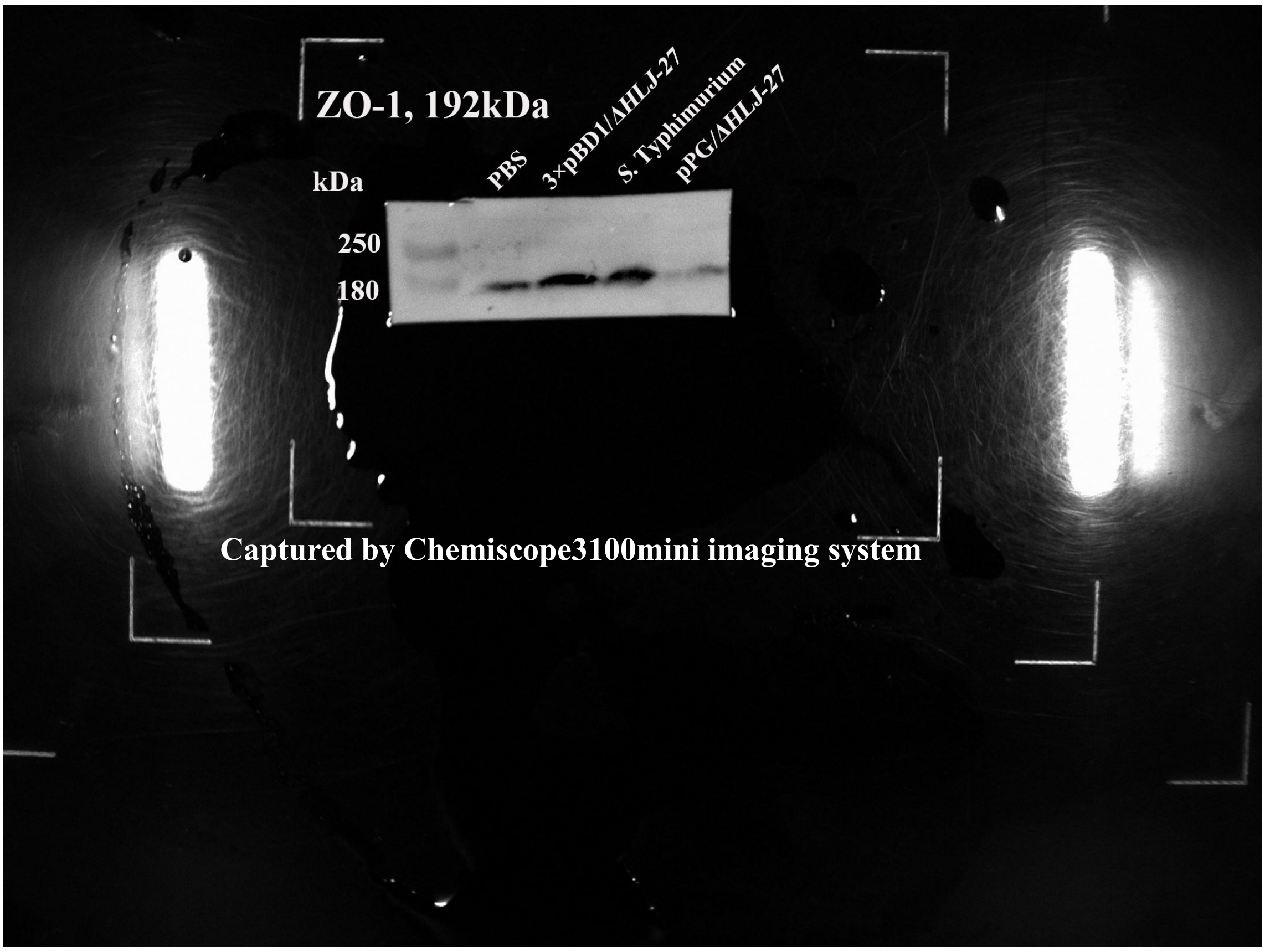
ZO-1, 192kDa
kDa
PBS
3×pBD1/ΔHLJ-27
S. Typhimurium
pPG/ΔHLJ-27
250
180
Captured by Chemiscope3100mini imaging system

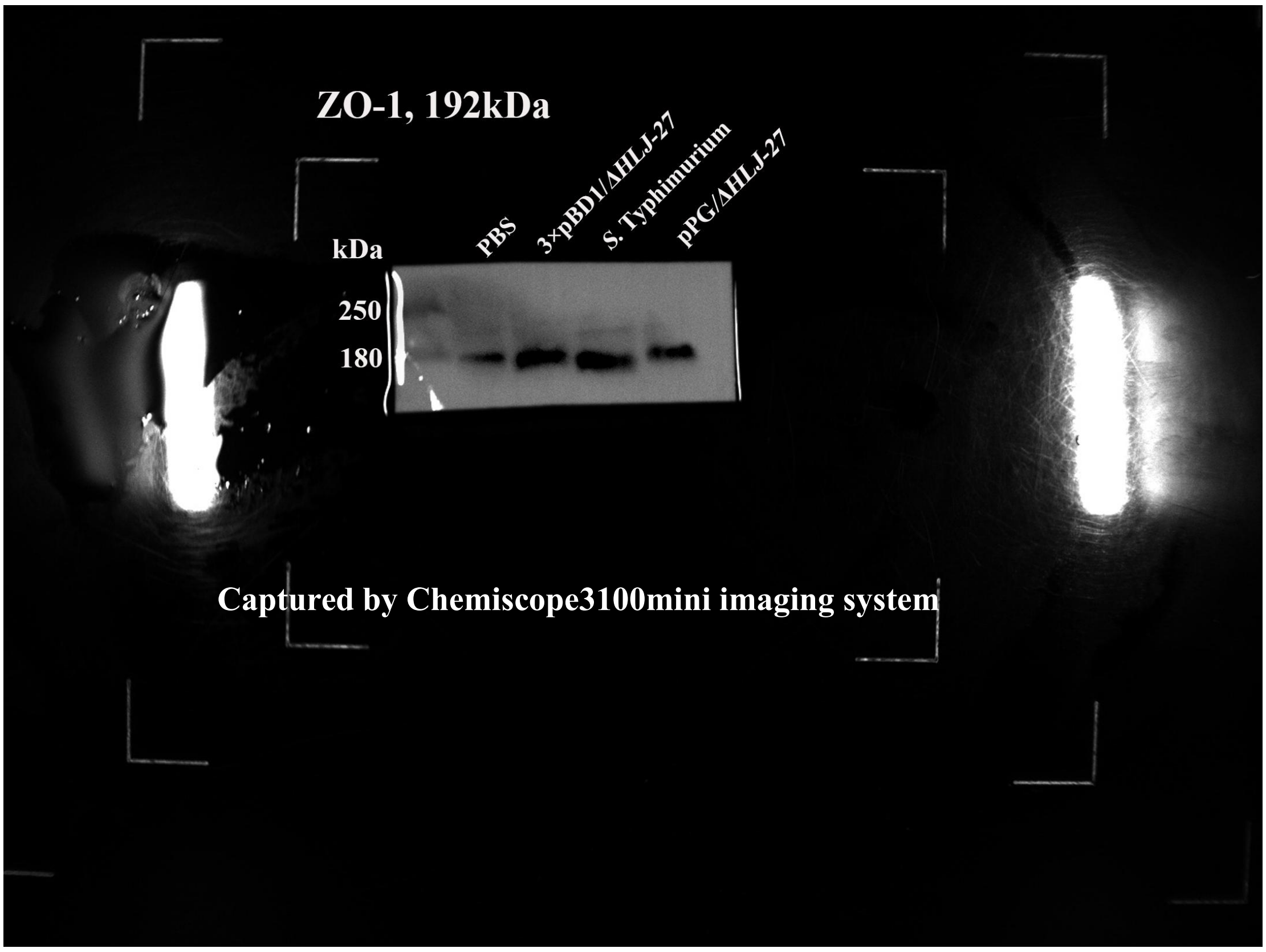
ZO-1, 192kDa
kDa
250
180
PBS
3×pBD1/ΔHLJ-27
S. Typhimurium
pPG/ΔHLJ-27
Captured by Chemiscope3100mini imaging system

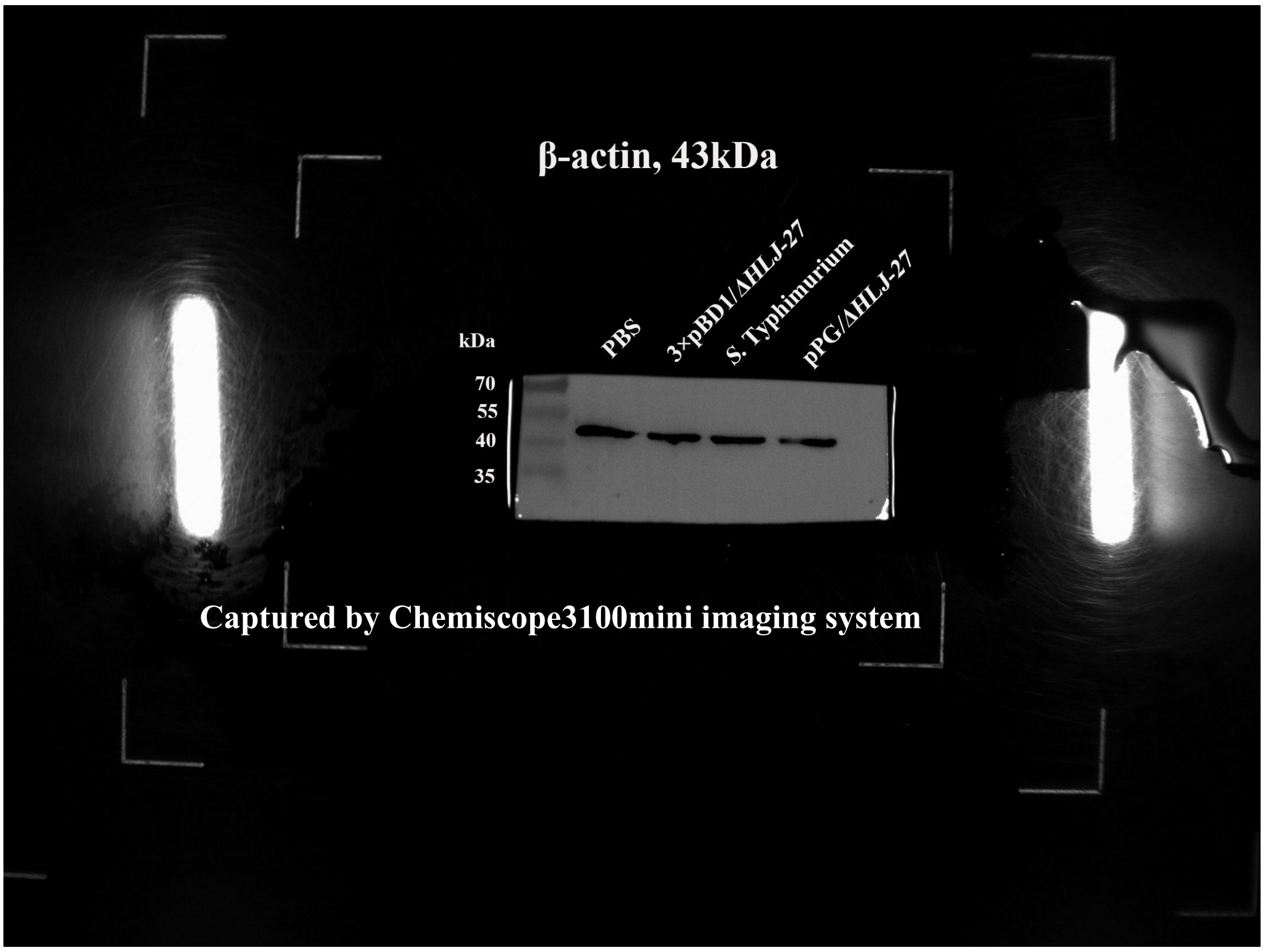
β-actin, 43kDa
kDa
70
55
40
35
PBS
3×pBD1/ΔHLJ-27
S. Typhimurium
pPG/ΔHLJ-27
Captured by Chemiscope3100mini imaging system

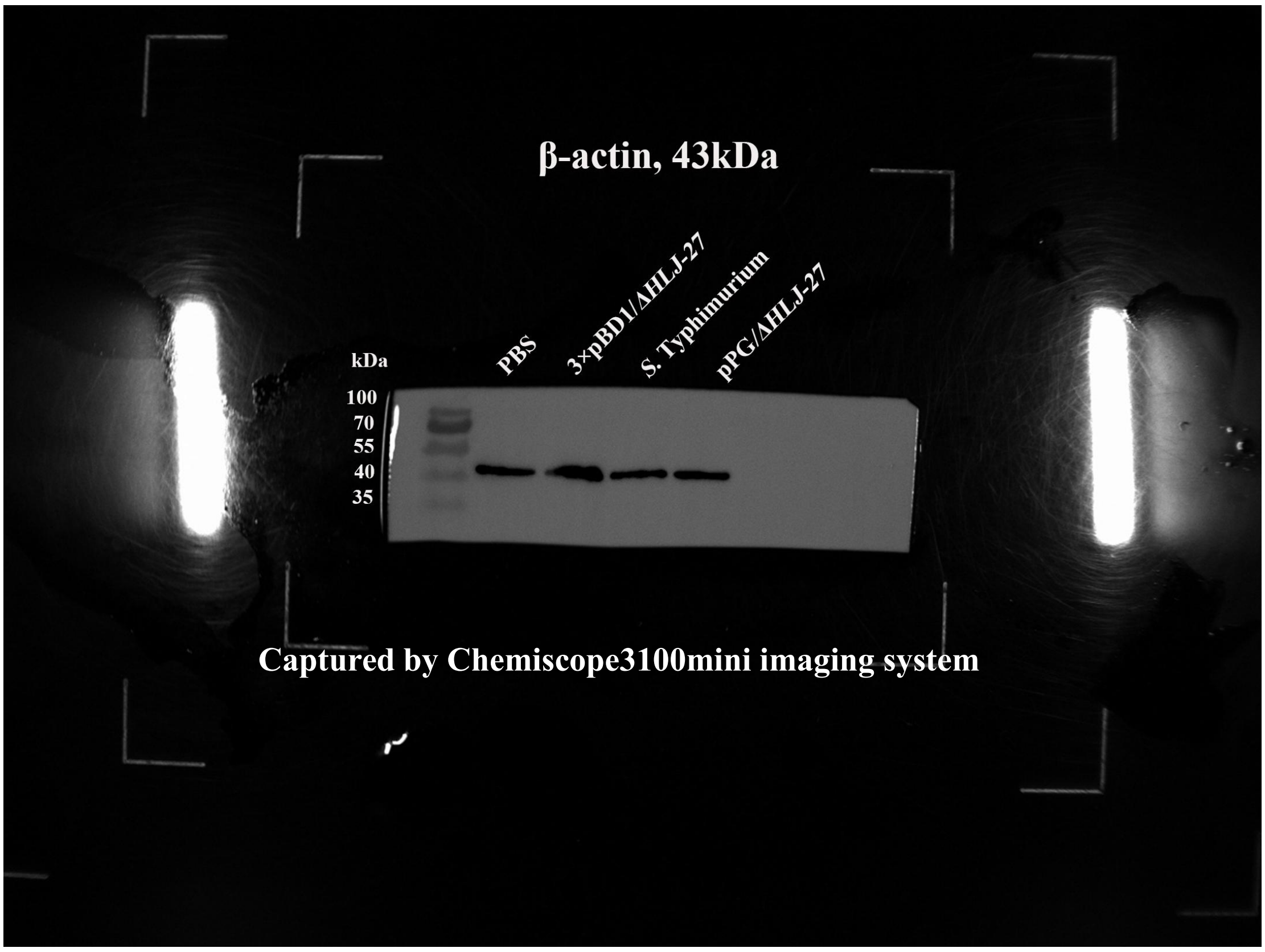

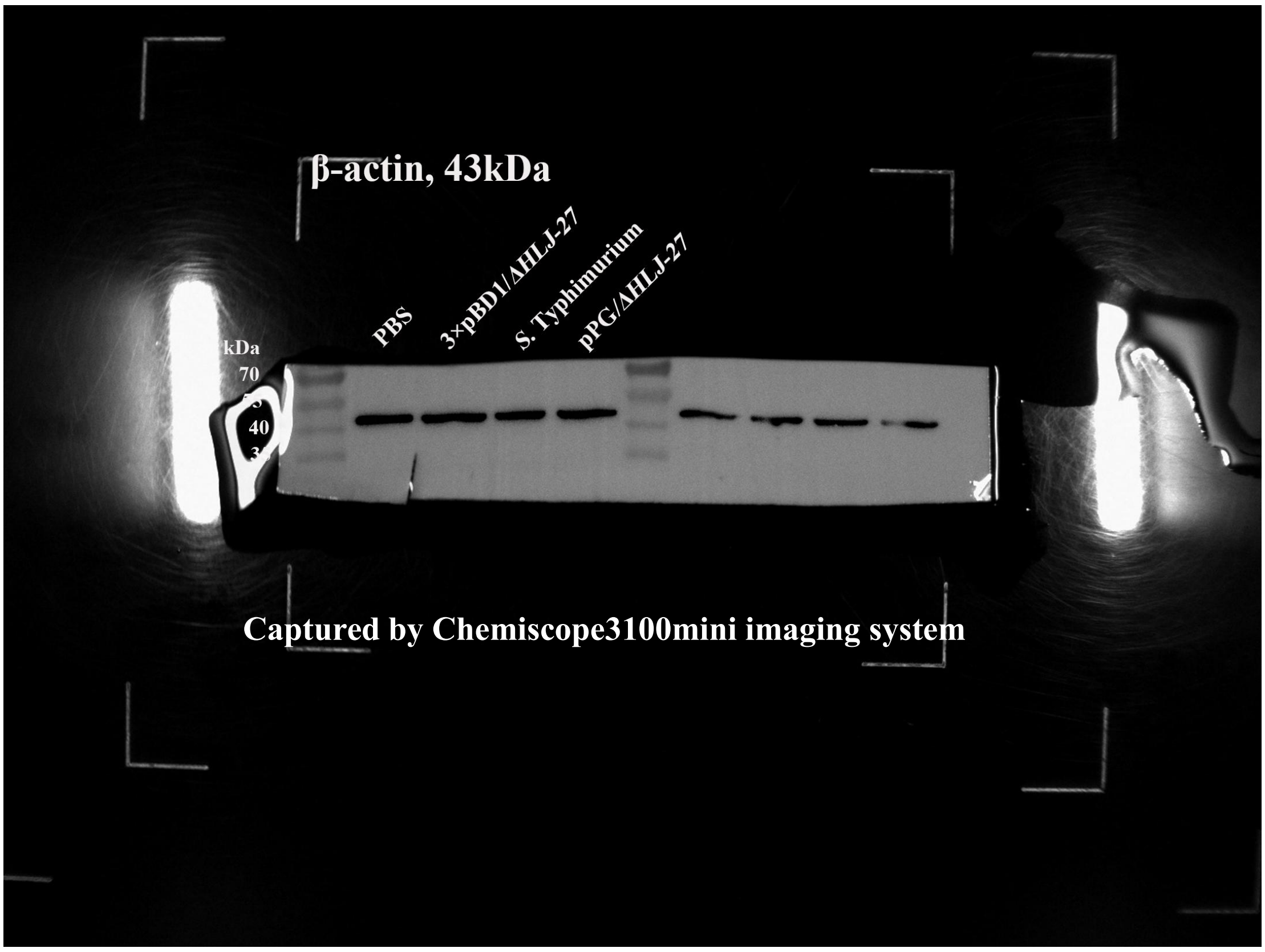

S1 Raw Image. Uncropped original Western blot membranes. This file contains uncropped original Western blot membranes corresponding to the protein band data shown in Figure 1G and Figure 7C of the main manuscript. All images were captured without adjustments to brightness, contrast or exposure.
