# Supplementary material for: Engineered Lactobacillus paracasei 3×pBD1/ΔHLJ-27 as a novel antibiotic alternative for livestock: Dual efficacy in growth promotion and protection against Salmonella infection via integrated host-microbiota-immune regulation
Source: PLoS Pathog. 2026 Aug 14;22(8):e1014367. doi: 10.1371/journal.ppat.1014367 (PMC13475985; doi:10.1371/journal.ppat.1014367)
Supplement: Supplementary file 2 [file ppat.1014367.s004.docx]

Table. S1 Primer sequence

| Gene | primer sequence |
| --- | --- |
| ZO-1 | F：CTGTGTGGGGCCTGACAA  R：AGTGCTTGCAGTCGAACTCA |
| Claudin-2 | F：ATGCCAGAGTAAATGTAC  R：CCACGTAGTAGACGATGGGC |
| Occludin | F：ACTGGGTCAGGGAATATCCA |
|  | R：TCAGCAGCAGCCATGTACTC |
| β-actin | F：TTCCTTCTTGGGTATGGAAT |
|  | R：GAGCAATGATCTTGATCTTC |
