# Supplementary material for: Engineered Lactobacillus paracasei 3×pBD1/ΔHLJ-27 as a novel antibiotic alternative for livestock: Dual efficacy in growth promotion and protection against Salmonella infection via integrated host-microbiota-immune regulation
Source: PLoS Pathog. 2026 Aug 14;22(8):e1014367. doi: 10.1371/journal.ppat.1014367 (PMC13475985; doi:10.1371/journal.ppat.1014367)
Supplement: Supplementary file 3 [file ppat.1014367.s005.docx]

Table. S2 Primer sequence

| Gene | primer sequence |
| --- | --- |
| CD40 | F：CGTGCGGGGACTAACAAGA |
|  | R：CCAACAGGACGGCAAACA |
| CD86 | F：ATGGGACTGAGTAACATTCT |
|  | R：TTCCAAGTATATGGGTCGCACA |
| CD80 | F：ATGGGCCACACACTGAAGTGGGA |
|  | R：AAGTATGAGAACCGCACCTT |
| TLR-2 | F：ACCATTCCCCAGCGTTTCT  R：GAGTCAGCAAGTCACCCTTTATGTT |
| TLR-4 | F：ACCAGACTTTCTTGCAGTGGGTCA  R：AATGACGGCCTCCCTTATCTGACA |
| TLR-6 | F：TCCCAGAATAGGATGCAGTGCCTT  R：ACTCCTTACATATGGGCAGGGCTT |
| S100A8 | F:CTGACGGATCTGGAGAGTGC  R:GGTTTCTGCGTCCTTTTTCTTCA |
| S100A9 | F:CCTGGGCTTGGAGAGAGAGG  R:TTTCTGGTGTGTGTGTCCC |
| CD14 | F:AGAGTGTAGCGAACGTGGG  R:TTTGGCAGACTTTGGGGGTT |
| TFF1 | F:CCCACAGACACAGAGGAGTG  R:AAGCAGTCCATCCACAGC |
| PLAU | F:CCACCCACTGCTTCATCAAT  R:ACGGATCTTCAGCAAGGCAA |
| ELOVL6 | F:CAAGAGCAAGGAGGTGGGAA  R:TCGCTACGTGTTCTCGAGTG |
| GSTM3 | F:TGTGCCGTTTTGAGGCTTTG  R:AGTCTCTTGTTGCCCCACTG |
